# Mass spectral and theoretical investigations of the transient proton-bound dimers on the cleavage processes of the peptide GHK and its analogues†

**DOI:** 10.1039/d0ra07600g

**Published:** 2021-01-20

**Authors:** Jinhu Wang, Cheng Wang, Han Zhang, Yang Liu, Tiesheng Shi

**Affiliations:** College of Chemistry, Chemical Engineering and Materials Science, Zaozhuang University Zaozhuang 277160 Shandong Province P. R. China; Department of Traditional Chinese Medicine, Zaozhuang Municipal Hospital Zaozhuang 277102 Shandong Province P. R. China wangjinhu@uzz.edu.cn

## Abstract

Fragmentation mechanisms of the singly protonated peptides GHK, GHKH and HGHK have been investigated by mass spectrometry and theoretical calculations. Fragmentation behavior of the protonated H–K amide bond in GHK was changed completely when a histidinyl residue was introduced into the C-terminus of GHK. The H–K amide bond breaking was a predominant pathway in the case of GHK and GHKH. For HGHK, the histidinyl residue at the N-terminus hampered significantly breaking of the H–K amide bond resulting in a high potential energy barrier; calculations indicated that this histidinyl effect played a vital role for the H–K amide bond fragmentation. Subsequent analysis of the fragmentation mechanism revealed that recombination processes of the hydrogen bonding for the intermediate products were all exergonic. Formation of a proton-bound dimer (PBD) lowering the energy barriers from a thermodynamic perspective for all the designed fragmentation pathways was demonstrated to be feasible by our systematic calculations. Moreover, the involvement of different PBDs was further confirmed by analyses of the reduced density gradient (RDG) isosurfaces and scatter maps. A dynamically favored pathway was likely *via* six-membered ring or nine-membered ring structures generated by the diketopiperazine as revealed by atom-in-molecules (AIM) analyses, since the steric interaction energies in the newly formed ring were estimated to be relatively small when compared to the products generated from a lactam and/or an oxazolone pathway. This is the first feasibility investigation from a dynamic viewpoint for formation of different rings involved in the lactam, oxazolone or diketopiperazine pathways in the fragmentation mechanisms proposed.

## Abstract 2

Fragmentation mechanisms of the singly protonated peptides GHK, GHKH and HGHK have been investigated by mass spectrometry and theoretical calculations.

## Graphical abstract

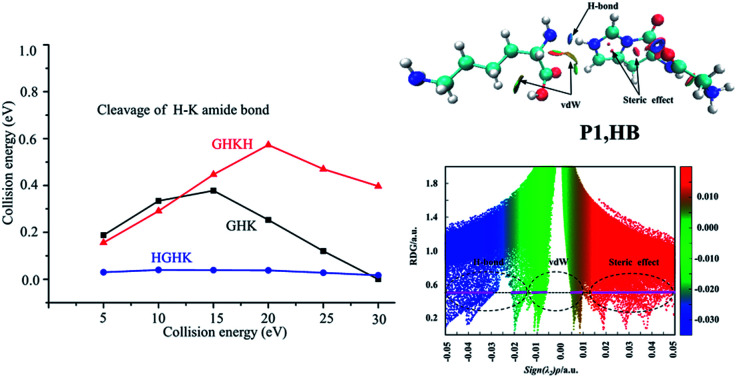

## 1. Introduction

Histidine (His) is an essential amino acid that belongs to the group of aromatic and heterocyclic amino acids.^1^ His-containing peptide GHK is known as a wound healing factor.^2^ It is normally present in human plasma, saliva, and urine, and it stimulates collagen, dermatan sulfate, chondroitin sulfate, and the small proteoglycan, decorin.^3^ GHK also enhances osteogenesis of cord blood mesenchymal stem cells, induces stem cell osteogenic differentiation,^4^ and participates actively in the processes of wound healing and tissue repair.^5^ The wound healing and antiaging properties of GHK are due probably to its strong affinity for copper ions (Cu^2+^) to form GHK–Cu complexes.^6–10^

Except for formation of the GHK–Cu complexes, protonation is also favored for GHK since the proton affinity of the His side chain is high.^11–13^ Experimentally and theoretically, there is compelling evidence for formation of nondirectly sequential ions *via* cyclization/reopening chemistry in the collision-induced dissociation (CID) spectra of the b_n_ ions when the His residue is near the C-terminus of peptides.^14^ The MS/MS spectra of the protonated peptides (HAAAAA, AHAAAA, AAHAAA, AAAHAA, and AAAAHA) show a series of b_n_ ions with a enhanced cleavage at the amide bond of the C-terminus to His, rendering a histidinyl effect.^14^

**Scheme 1:**
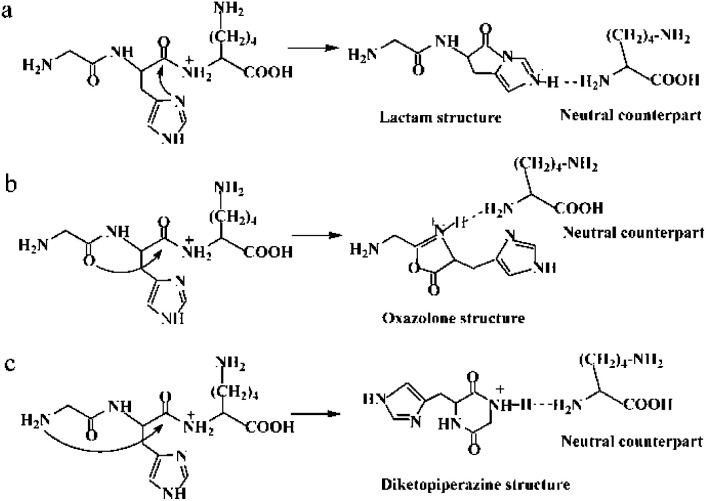
Possible mechanisms for the protonated GHK leading to amide bond fragmentation: (a) lactam pathway; (b) oxazolone pathway; (c) diketopiperazine pathway.

Histidinyl effect originates from a mobilization of the proton located at the His side chain to the nitrogen of the C-terminally neighboured amide bond.^11–13^ A subsequent nucleophilic attack of the imidazolyl nitrogen on the carbon of the protonated amide bond can generate a transient lactam structure, which is a non-classical oxazolone derivative (Path a in Scheme 1). Moreover, an isomerization from the transient lactam to an oxazolone is potentially possible.^15^ Formation of the well-known oxazolone involves a nucleophilic attack of backbone amide oxygen (the N-terminally neighboured amide bond) on the C-terminally adjacent nitrogen of the protonated amide bond (Path b in Scheme 1).^16^ Several studies have suggested that the oxazolone structure is likely a b_2_ isomer.^17–20^ Moreover, the possibility for an isomerization between an oxazolone and a diketopiperazine form has been exploited.^21^ In the diketopiperazine pathway, the N-terminal amino group acts as a nucleophile and attacks the carbonyl carbon of the second amino acid residue, forming a more stable six-membered ring structure (Path c in Scheme 1).^22^ Such a cyclized ion involves a *trans*–*cis* isomerization of the amide bond.^13^ The fragmentation patterns of His-containing b_2_ ions from the protonated HG–OMe, GH–OMe, HGG–OMe, and GHG–OMe were compared with the diketopiperazine structure generated from dipeptide GH and very similar fragmentation spectra were found, supporting the formation of a diketopiperazine (cyclic dipeptide) b_2_ ion.^12^

A spectroscopic study of protonated tripeptide AAA and a cyclo dipeptide (cyclo-AA) by infrared multiple photon dissociation (IRMPD) technique indicated that the product is not a diketopiperazine, rather an oxazolone protonated at the oxazolone N-atom.^23^ Another IRMPD study of the protonated peptide HAAAA and cyclic dipeptide cyclo (HA) provided the first spectroscopic evidence for the presence of a mixture of oxazolone and diketopiperazine.^24^ A jointly multistage mass spectral and *ab initio* study demonstrated that the cyclic structures involving the side chains of Arg, His, and Lys, are more stable than the oxazolone ones.^25^

A study of His-containing peptides including FH–OMe, FH–NH_2_, FHA, HF–OMe, HF–NH_2_ and HFA by energy-resolved electrospray/Quadrupole/Time-of-Flight (QqToF) and by Density Functional Theory (DFT) calculations showed that the position of the His residue indeed influences the identity of the subsequent b_2_ ion, where the leaving group significantly affects the transition state energies and structures of the b_2_ ion.^21^ Under the conditions of low-energy collisions for the mass spectral measurements, the loose complex of the protonated oxazolone derivative and the leaving C-terminal fragment could undergo a rearrangement, resulting in formation of a proton-bound dimer (PBD) (see Scheme 1).^26^ Furthermore, under the low-energy conditions, the lifetime of the dimer was long enough so that various PBD isomers could interconvert^27^ and proton transfers took place between the proton donors and acceptors.^28,29^

The separation of PBD was the final step in most of the fragmentation mechanisms proposed for the protonated peptides.^15^ However, most investigations were focusing only on the products of charged ions (such as lactam, oxazolone or diketopiperazine ions), where the leaving groups (neutral counterparts) were not investigated systematically. The structure of neutral counterparts affects the energies of the dissociation reaction (as does the structure of the product ion), which in turn has a major impact on the preferred dissociation pathway and the structure of the product ion. Occasionally, neutral counterparts of charged ions could attract a proton and form a new charged ion.^13,30^ When only a PBD was formed, the followed dissociation reactions possibly generated b_*x*_ or y_*z*_ ions.^15^

In this work, the fragmentation reactions were investigated involving some leaving groups and formation of PBDs which are labeled by Pn′ in the text. The fragmentations of protonated GHK and of its analogues GHKH and HGHK were analyzed for examinations of the influence of His residue. All the three pathways described in Scheme 1 contain different dissociation channels, leading to formation of a mixture of three isomeric forms of the N-terminus fragments. Since the PBD was vital in the formation of b_*x*_ or y_*z*_ ions, cleavage processes of the peptide GHK and its analogues (GHKH and HGHK) were studied. As the Lac pathway only took place on the amide bond of H–K for our subjects, the cleavage of H–K amide bond was selected as the cleavage amide bond for the experimental and theoretical study in the following Lac, Dik and Oxa pathways. The identification of the intermediates in these pathways bolsters the dissociation mechanisms, enabling us to have a deeper understanding of the cleavage behavior of H–K amide bond in these peptides.

## 2. Experimental and theoretical methods

### 2.1 Experimental section

The peptides GHK, GHKH and HGHK with a purity ≥ 95% were purchased from Guoping Pharmaceutical Inc. (Hefei, China); they were used without further purification. Stock solutions of 0.1 mM peptides were prepared by dissolving desired amounts of the peptides in a solvent mixture of water and methanol (in a ratio of 1 : 1 (v/v) containing 0.5% formic acid); HPLC-grade methanol and ultrapure deionized water (>18.2 M Ω cm^−1^) were used as the solvents.

The stock solutions were diluted to 20 μM by water, and then directly infused, at a rate of 15 μL min^−1^, into the capillary of an electrospray ionization (ESI) tandem mass spectrometer (LTQ Orbitrap Velos, Thermo Scientific, San Jose, CA, USA); methanol was used as the mobile phase. Electrospray and ion focusing conditions were adjusted to maximize the relative abundance of the singly protonated peptides and fragmented ions. Full scan mass spectra with a mass window of 1000 Da were acquired after an accumulation of about 1 × 10^6^ counts in the positive mode. Helium was used as the collision gas for the ion trap at a pressure of 2.05 × 10^−5^ torr. Protonated peptides subjected to low energies of CID yield the complementary b_*n*_ and y_*n*_ series of sequence ions in the trap cell, where the ESI parameters were optimized to be: electrode needle voltage of 3500 V, sample cone voltage of 55 V, extraction cone voltage of 0.50 V, source temperature of 110 °C, and cone nitrogen gas flow rate of 30 L h^−1^. Mass number deviation was set at 100 ppm, where the delta of retention time was adopted the default value of 0.5 min.

### 2.2 Theoretical methods

#### 2.2.1 Geometric optimizations

Geometric optimizations for the neutral structures of GHK, GHKH and HGHK were performed at the B3LYP/def-TZVP level using the Turbomole module program.^31^ Based on the optimized structures, the singly protonated peptides of GHK, GHKH and HGHK were then generated with a mobile proton at the designed positions. Molecular Dynamics (MD) simulation at 1000 ps in gas phase was performed to achieve an equilibration using the CHARMM22 force field^32^ in CHARMM C43B1.^33^ Numerous structures from the MD trajectory were sampled regularly and a series of calculations of single point energy were performed to find the structures with reliably low energies, as shown in Fig. S1 in ESI.† The structure with the lowest energy was selected to perform a geometric optimization and to search reaction pathways^18^ at the B3LYP/def-TZVP level using Turbomole supported by ChemShell 3.7.0.^34^ Key transition states with only one imaginary frequency were further validated by intrinsic reaction coordinate (IRC) calculations to confirm their connections.^35–37^

#### 2.2.2 Analysis by atoms-in-molecules (AIM)

The AIM theory has been demonstrated to be a very useful approach to explore non-covalent bonding interactions employing electron density and laplacian at the critical points with the utilization of the optimized structures.^38^ Hydrogen bonding energies (*E*_HB_) were derived from the potentials of electron energy density (*V*_(r)_) according to *E*_HB_ = *V*_(r)_/2.^39^ All possibly critical points were determined and visualized using the Multiwfn program 3.5.^40^

Since the electron densities in the non-covalent bonding regions are relatively low, examination of the non-covalent bonding interactions by evaluating the reduced density gradient (RDG) functions is a reliable approach.^41^ Secondary Hessian eigenvalue (*λ*_2_) varies in sign (sign(*λ*_2_)*ρ*) depending on a bonding nature, thus sign(*λ*_2_)ρ can be used as an indicator to distinguish the interactions of non-bonding (when sign(*λ*_2_)*ρ* > 0) from bonding (when sign(*λ*_2_)*ρ* < 0).^42^ Molecular structures and non-covalent bonding interactions were directly visualized and quantified from the analysis of 3-D RDG isosurfaces when the sign(*λ*_2_)*ρ* values were encoded with different colours.^41^ The analysis of 3-D RDG isosurfaces was also based on the optimized chemical structures^38^ resembling the AIM approach.

## 3. Results and discussion

### 3.1 Mass spectra

#### 3.1.1 Mass spectra of singly protonated GHK

**Fig. 1:**
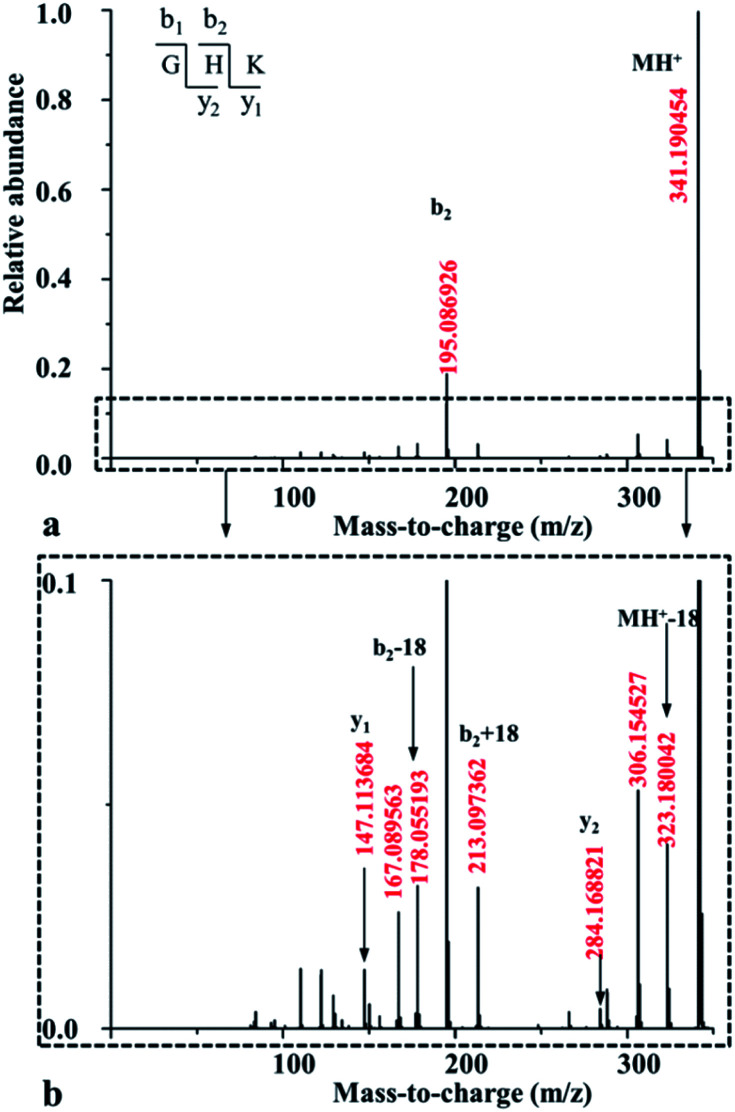
MS/MS spectrum acquired for the singly protonated GHK at the potential of 5.0 eV (a), together with its enlarged one in *y*-axis (b).

The mass spectra of singly protonated GHK were recorded under different potentials of CID ranging from 5 to 30 eV, which are given in Fig. S2 in the ESI.† The mass spectrum acquired at 5.0 eV is shown in Fig. 1, which contains most of the sequentially-informative b_2_, y_1_ and y_2_-type fragments. The mass spectra obtained at higher CID potentials become less informative (see Fig. S2 in the ESI†). The mass peak of *m*/*z* 341.190 in Fig. 1 is the precursor ion (namely MH^+^) for singly protonated GHK; the protonation site is presumably at the His side chain owing to its high proton affinity.^13^ However, the initial protonation site will not initiate a fragmentation of the amide bonds, thus a fraction of protons rapidly transfer to the energetically less favourable nitrogen atoms of the backbone amides, leading to amide bond cleavages. The dominant peak of *m*/*z* 195.087 is clearly formed from the H–K amide bond cleavage as a b_2_-type ion. It might couple with a nucleophilic attack of the imidazolyl nitrogen on the carbon of the protonated amide bond (Path a in Scheme 1), resulting in formation of a non-oxazolone derivative. In fact, Paths b and c in Scheme 1 generate different b_2_ ions. The y_1_-type ion derived from the H–K amide bond cleavage is also detectable although the abundance is much lower than that of the accompanying b_2_ ion, indicating that the cleavage of H–K amide bond mainly produces a b_2_ ion.

#### 3.1.2 Fragmentation pathway of the singly protonated GHKH and HGHK

Mass spectra of the singly protonated GHKH and HGHK were recorded under different CID potentials ranging from 5 to 30 eV, which are summarized in Fig. S3 and S4 in the ESI.† Main fragment ions of the protonated GHKH and HGHK are listed in Table 1.

**Table 1:** Fragmentation of protonated GHKH and HGHK using the CID potential energy of 5 eV^a^

Precursor	Fragment	*m*/*z*	Relative abundance (%)
GHKH	b_2_	195.085	15.6
	b_3_	323.178	13.5
	y_1_	156.070	6.6
	MH^+^-18-17	443.216	6.2
	MH^+^	478.248	99.9
HGHK	a_1_	110.073	7.5
	b_2_	195.084	7.9
	b_3_	323.150	2.1
	y_2_	284.167	7.2
	y_3_	341.190	3.0
	MH^+^-18	460.235	4.3
	MH^+^	478.251	99.9

a (1) MH^+^ represents the precursor ion for singly protonated peptides; (2) relative abundances presented here were obtained in relative to the abundance of precursor ion at CID 5 eV.

For both GHKH and HGHK, the precursor ions (MH^+^ in Table 1) are all at a relative abundance of 99.9%; only a small percentage of precursor ions fragments at low energies, base on which we can judge which amide bond is easily cleavage. Apart from the loss of a water and/or an ammonia (MH^+^-18-17 for GHKH and MH-18 for HGHK), other site cleavages occurred entirely at the backbone amide bonds at CID potential of 5 eV. The mass peak at *m*/*z* 478.248 is a precursor ion for the protonated GHKH. Similar to protonated GHK, the relative abundance of the b_2_-type fragment ion (*m*/*z* 195.085) is increased to 15.6%, indicating that a cleavage of the H–K amide bond is preferred. Obviously, the addition of an His residue to the C-terminus of GHK does not hamper the H–K amide bond cleavage. Thus, all the three fragmentation pathways described in Scheme 1 are all possible to occur rendering a formation of the b_2_ ion similar to the case of the protonated GHK. Another peak at *m*/*z* 323.178 is assigned to the truncated GHK fragment as the b_3_-type ion, being generated by a cleavage of the K–H amide bond. The y_1_-type fragment ion derived from the K–H amide bond cleavage is also detectable. Comparing with that of b_3_ ion, the strength of this ion is relatively lower, suggesting that b_3_ is the dominant ion for the K–H amide bond cleavage. On the other hand, the cleavage at the N-terminal side of His from the G–H amide bond is not detectable.

The mass peak at *m*/*z* 478.251 in Table 1 corresponds to the protonated ion of HGHK. A dominant peak at *m*/*z* 110.073, differentiating from those of the protonated GHK and GHKH, is a secondary fragmentation product from an amide cleavage by loss of a CO (a b_1_ ion), being assigned to an a_1_-type fragment ion. Fragmentation efficiencies *versus* the applied collision energies for H–G amide bond are given in Fig. S5 in ESI.† Clearly, the collision energies have a significant influence on the H–G amide bond fragmentations, *i.e.*, the higher of the energies, the stronger of the relative abundance of the a_1_-type fragment ions. Hence, the His effect determines if the cleavage at the C-terminus of His and the a_1_ ion is generated. The protonated GHK and GHKH did not produce a_1_ ions since GHK and GHKH possess the same N-terminal Gly and the charge cannot be stabilized by N-terminal fragment after cleavage. However, when an His residue is at the N-terminus, and the charge can be fixed by the side chain of the His.

Another major peak at *m*/*z* 195.085 is formed from the G–H amide bond cleavage which is assigned to a b_2_-type fragment ion being produced by the cleavage at the N-terminal His. Mass peak at *m*/*z* 284.167 is assigned to the truncated HK fragment as an y_2_-type ion, being generated from the cleavage of the G–H amide bond. A b_3_-type fragment ion derived from the H–K amide bond cleavage is also detectable. The strength of b_3_ ion is relatively lower compared with that of the b_2_ ion. It is thus concluded that the His residue at N-terminus can prohibit cleavage of the backbone H–K amide bond.

#### 3.1.3 Influence of the His residue on the H–K amide bond fragmentation

**Fig. 2:**
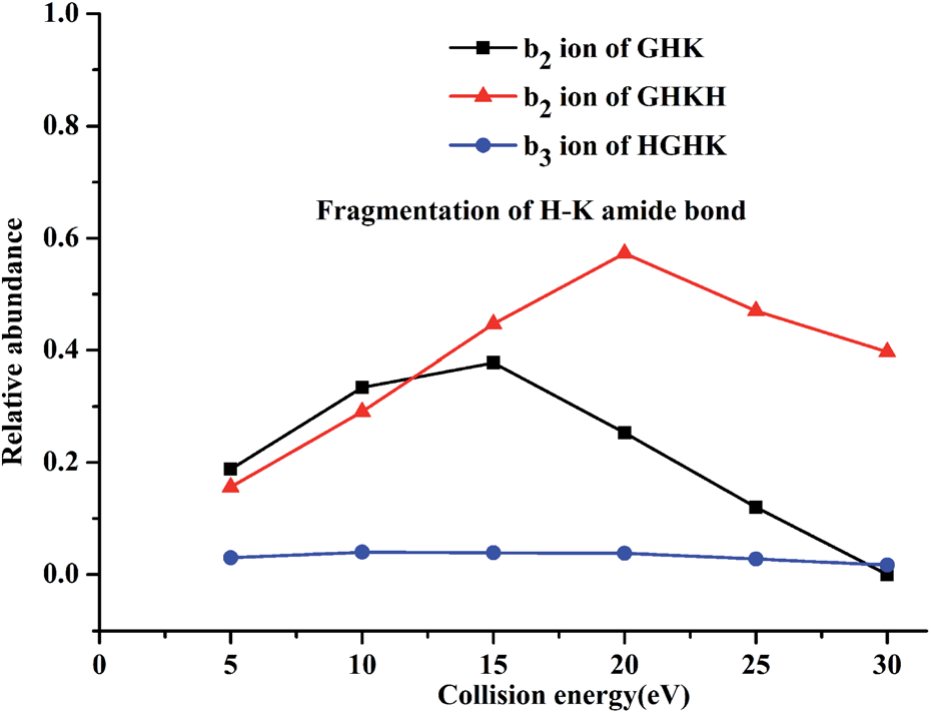
Relative abundances for the H–K amide bond fragmentations for the singly protonated peptidyl ions of GHK, GHKH and HGHK.

Fragmentation efficiencies *versus* the applied collision energies for H–K amide bond are shown in Fig. 2. Apparently, the collision energies have a significant influence in the cases of GHK and GHKH. The abundance of the b_2_ ion for the protonated GHK increases until the energy reaches 15 eV, where the same type of ion for the protonated GHKH increases until the energy goes up to 20 eV. As to the b_3_ ion of HGHK, the energies almost have no influence on the H–K amide bond fragmentation. Analyses of fragment ions for GHKH and HGHK show that the cleavage of the H–K bond becomes easier when a His residue is attached to the C-terminus, where the cleavage of H–K bond becomes less easy when a His residue is located at the N-terminus.

### 3.2 Theoretical calculations

#### 3.2.1 Analysis of H–K amide bond fragmentation of the protonated GHK

**Scheme 2:**
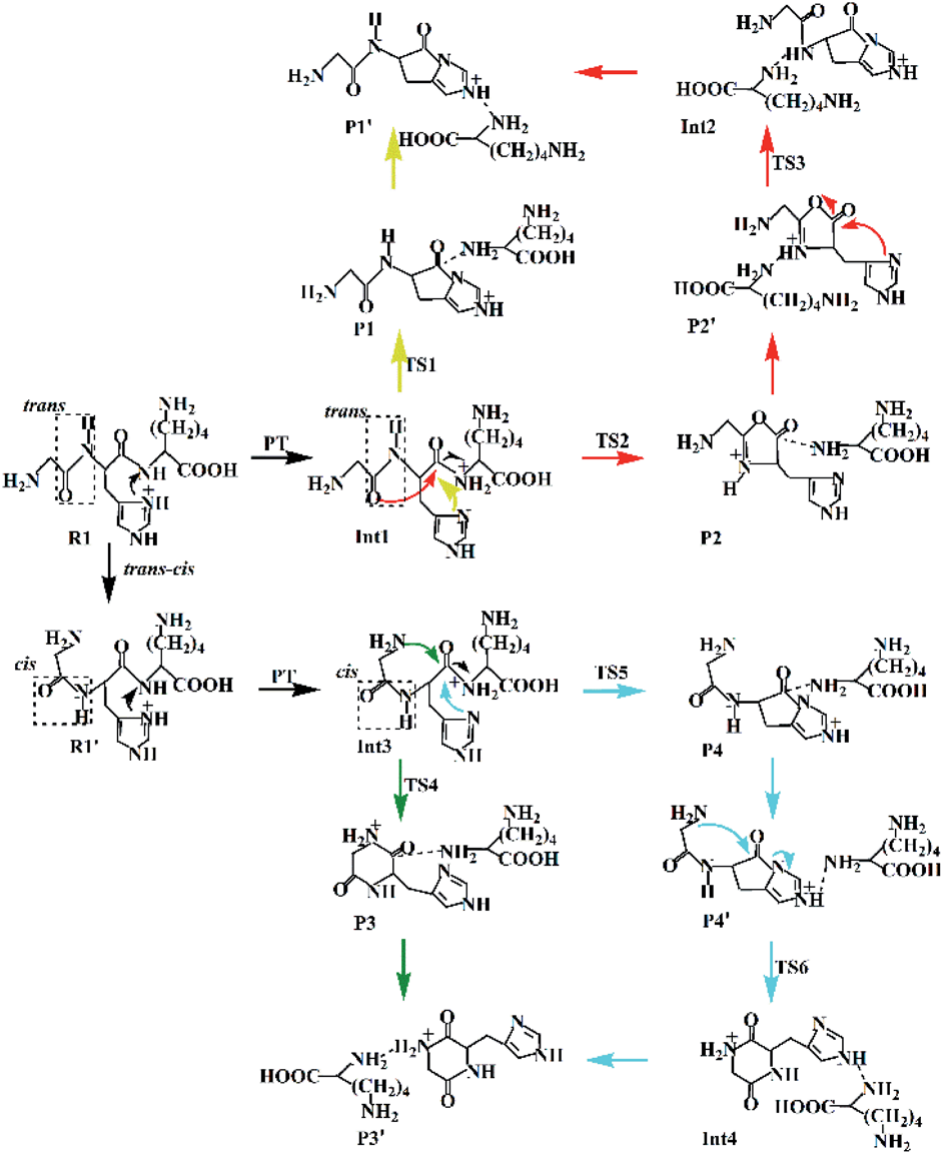
Proposed fragmentation mechanism for the protonated GHK.

A plausible mechanism proposed for the H–K amide bond fragmentation of the protonated GHK ion is depicted in Scheme 2 including four possible cyclization reactions. The first one is involved in the lactam pathway, proceeding *via* a nucleophilic attack on the second amide carbonyl carbon by the imidazolyl nitrogen of the His (yellow line, named as Lac pathway). The second one is of a combined oxazolone–lactam (Oxa–Lac) pathway, starting by a nucleophilic attack of the N-terminally adjacent carbonyl oxygen on the amide carbonyl carbon, followed by a lactam pathway (red line, Oxa–Lac pathway). No *trans*–*cis* isomerization is expected to occur for the G–H amide bond in the above two pathways. The third one is formed by a nucleophilic attack of the N-terminal amine group on the amide carbonyl carbon (green line, Dik pathway). A combined mechanism of lactam and diketopiperazine is also possible (cyan line, Lac–Dik pathway). The *trans*–*cis* isomerization of the G–H amide bond occurs firstly for both the Dik and Lac–Dik pathways.

Reaction coordinates of all the optimized structures for the protonated GHK and its analogues are listed in Fig. S6–S16 in the ESI† while energies of all the optimized structures are given in Tables S1–S3 in the ESI.† Energy profiles of the designed reaction pathways are shown in Fig. S17–S19 in the ESI,† and the major optimized transition states for the H–K amide bond cleavage are given in Fig. S20–S22 in the ESI.† These major transition states are further validated by IRC calculations and are shown in Fig. S23–S25 in the ESI.†

##### (a) The lactam direct pathway

**Fig. 3:**
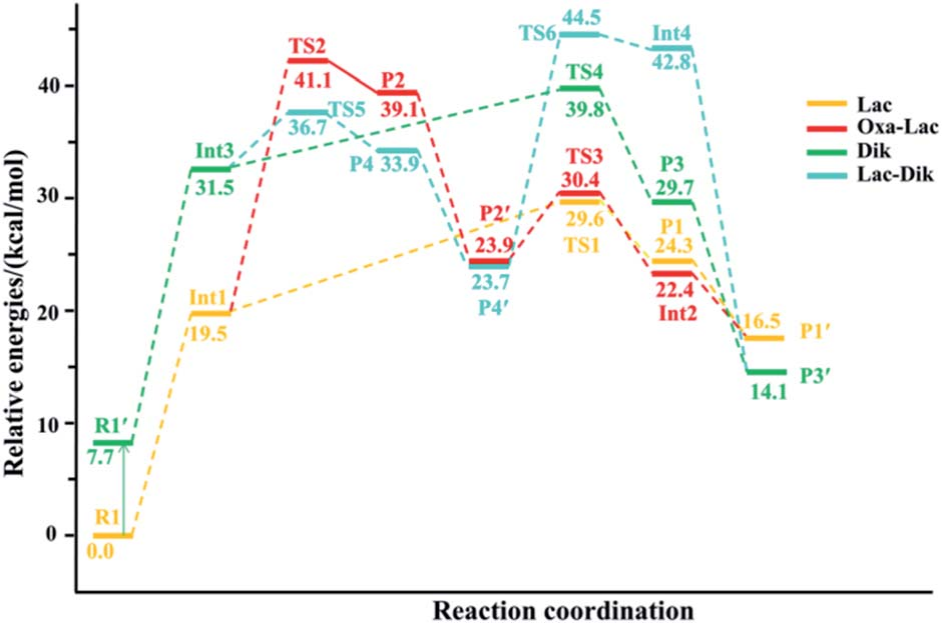
Energy profile for the H–K amide bond cleavage of the protonated GHK.

In the lactam pathway, reactant R1 firstly undergoes a proton transfer from the His side chain to the H–K amide bond oxygen, where the intermediate Int1 is possibly formed. The cleavage of the carbonyl carbon to the H–K amide nitrogen occurs consecutively. The C–N amide bond cleavage directly affords the product P1*via* the transition state TS1. This product can generate a PBD (P1′). The barrier to the H–K amide bond breaking from Int1 to P1 is calculated to be 29.6 kcal mol^−1^ (yellow line in Fig. 3). The formation of P1′ for the N- and C-terminal fragments (P1→P1′) readily takes place since P1′ is 7.8 kcal mol^−1^ lower in energy than that of the product P1.

Two nucleophilic attacks may occur sequentially for Int1 in the combined Oxa–Lac pathway, *cf.*Scheme 2. One attack takes place by the N-terminally neighboured amide oxygen on the carbon centre of the protonated amide bond, breaking the C-terminally neighboured amide bond and affording the oxazolone product P2*via* the transition state TS2. This needs to overcome an energy barrier of 41.1 kcal mol^−1^ (the red line in Fig. 3). After the recombination of the hydrogen bonding model, a favoured product of P2′ is generated, and it is exergonic by 15.2 kcal mol^−1^ relative to P2. The other attack occurs by the imidazolyl nitrogen of the His on the amide carbonyl carbon, giving rise to the lactam intermediate Int2*via* the transition state TS3 and requiring to overcome an energy barrier of 30.4 kcal mol^−1^.

##### (b) The diketopiperazine direct pathway

Starting from R1, the *trans*–*cis* isomerization of the N-terminal G–H amide bond gives the potential intermediate R1′ (green line in Scheme 2) with an endothermic energy of 7.7 kcal mol^−1^ (green line in Fig. 3). Thus, structures generated from the diketopiperazine (green line) and the lactam-diketopiperazine (cyan line) pathways all have the *cis* G–H amide bond. The initial part of the diketopiperazine pathway also involves a proton transfer from R1′ to the intermediate Int3 requiring an energy of 23.8 kcal mol^−1^ (Fig. 3). Int3 then undergoes a cyclization by a nucleophilic attack of the N-terminal amine group on the amide carbonyl carbon (green line in Scheme 2) *via* the transition state TS4, yielding the diketopiperazine product P3 with an energy barrier of 39.8 kcal mol^−1^ (TS4 in Fig. 3). Subsequently, P3 recombines the hydrogen bonding model, and the favourable product P3′ is generated with an exergonic energy of 15.6 kcal mol^−1^ relative to P3.

##### (c) The combined lactam–diketopiperazine direct pathway

Starting from Int3, two nucleophilic attacks are proposed to occur sequentially in the combined Lac–Dik pathway shown in Scheme 2 (cyan line). A concerted nucleophilic attack of the imidazolyl nitrogen of the His on the carbon centre of the protonated amide bond proceeds firstly to afford the lactam product P4*via* the transition state TS5 by overcoming an energy barrier of 36.7 kcal mol^−1^ (the cyan line in Fig. 3). After the recombination of the hydrogen bonding model, the favourable product P4′ is generated with an exergonic energy of 10.2 kcal mol^−1^ relative to P4. This is followed by another nucleophilic attack of the N-terminal amine group on the amide carbonyl carbon, giving rise to the diketopiperazine intermediate Int4*via* the transition state TS6 with an energy barrier of 44.5 kcal mol^−1^.

The optimized transition states for protonated GHK are shown in Fig. S20 in the ESI.† The transition state TS1 is generated from the Lac pathway. The nucleophilic attack of the imidazolyl nitrogen of the His leads to an elongatation of the H–K amide bond to 2.00 Å. The electron rich imidazolyl nitrogen atom is about 1.56 Å away from the carbon of the protonated H–K amide bond. The optimized structures of TS2 and TS3 are obtained from the Oxa–Lac pathway. The nucleophilic attack makes the N-terminally adjacent carbonyl oxygen to be 1.59 Å away from the protonated amide carbon atom in TS2, where this kind of distance is 1.73 Å in TS3. The protonated amide bond is elongated to 2.35 Å in TS2, which is 0.35 Å longer than that in TS1. The transition state TS4 is achieved from the Dik pathway when the N-terminal amine group is 1.80 Å away from the amide carbonyl carbon, being accompanied with an elongation of the H–K amide bond to 1.77 Å. Another Lac–Dik pathway gives two transition states (TS5 and TS6). TS5 is obtained by an approach of the imidazolyl nitrogen of His onto the carbon centre of the protonated amide bond (distance of 1.53 Å), while TS6 is achieved by a nucleophilic attack of the N-terminal amine group on the amide carbonyl carbon (1.70 Å). Undoubtedly, the above nucleophilic attacks largely weaken the covalent bonds linking to the carbon centre of the protonated amide bond as shown by the bond lengths (2.21 and 1.94 Å for TS5 and TS6, respectively).

#### 3.2.2 Analysis of H–K amide bond fragmentation for the protonated GHKH

**Scheme 3:**
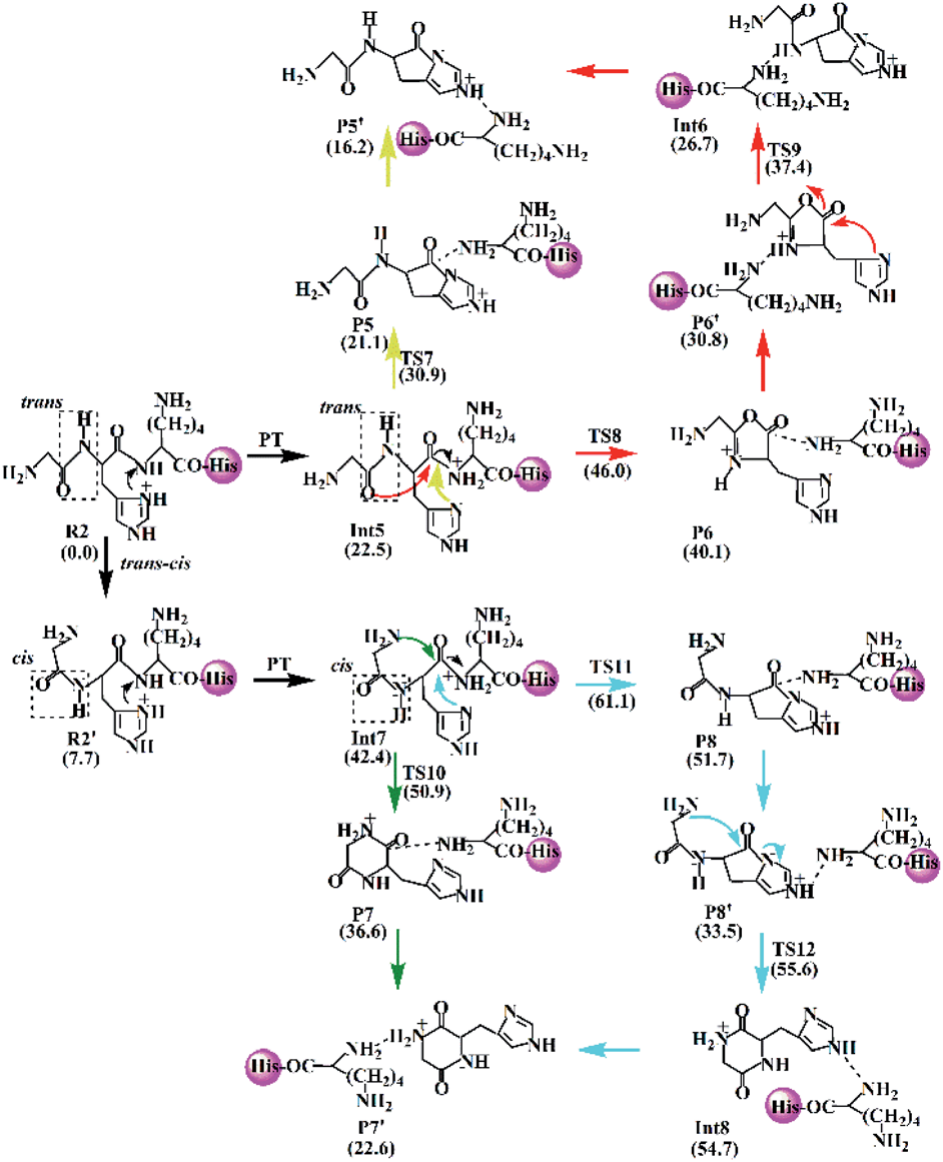
Proposed fragmentation mechanism for the protonated GHKH.

A mechanism for the cleavage of H–K amide bond is proposed and described in Scheme 3 for the protonated GHKH including the Lac pathway (yellow line), the combined Oxa–Lac pathway (red line), Dik pathway (green line), and the combined Lac–Dik pathway (cyan line). The mechanism is very similar to that proposed for the protonated GHK, encompassing similar cyclization reactions. In addition, the *trans*–*cis* isomerization of the G–H amide bond occurs firstly for both of Dik and Lac–Dik pathways. Scheme 3 involves the reactant R2, the intermediates Int5 and Int6, the transition states TS7–TS12, the product P5–P8, and the PBDs P5′–P8′. The energy barriers are marked in the Scheme. The optimized transition states for the protonated GHKH are shown in Fig. S21 in the ESI.†

#### 3.2.3 Analysis of H–K amide bond fragmentation for the protonated HGHK

**Scheme 4:**
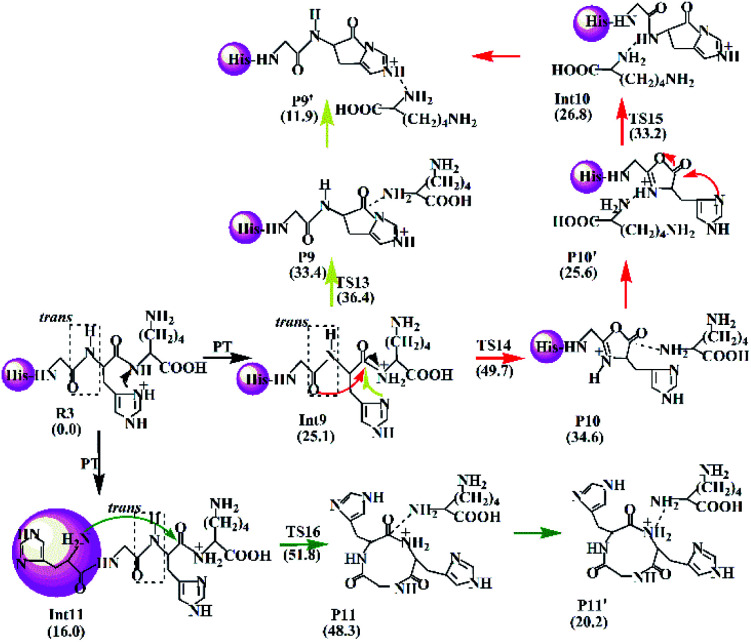
Proposed fragmentation mechanism for the protonated HGHK.

In the case of protonated HGHK, three possible pathways for the cleavage of H–K bond are suggested and described in Scheme 4 including Lac, Oxa–Lac, and Dik ones. Similar to the cases of protonated GHK and GHKH, the Lac and Oxa–Lac pathways involve nucleophilic attacks by the side chain (yellow line) and the N-terminally adjacent carbonyl oxygen (red line), respectively. A difference lies in the third pathway (the Dik one), where a nine-member ring is formed after a nucleophilic attack of the N-terminal amine group on the amide carbonyl carbon (green line), in contrast to the cases of protonated GHK and GHKH where a six-member ring is formed.

The Lac pathway involves the reactant R3, the intermediate Int9, the product P9, the transition state TS13 and the PBD P9′; the combined Oxa–Lac pathway encompasses the intermediate Int11, the product P11, the transition state TS15 and the PBD P10′; the Dik direct pathway consists of the intermediates Int9 and Int10, the product P10, the transition state TS16 and the PBD P11′. The energy barriers are also shown in Scheme 4. The optimized transition states for protonated HGHK are shown in Fig. S22 in the ESI.† In TS13, the nucleophilic attack of the imidazolyl nitrogen of the His leads to an elongation of the H–K amide bond to 1.73 Å. The electron-rich imidazolyl nitrogen atom is about 1.69 Å away from the carbon of the protonated H–K amide bond. In TS14, the nucleophilic attack makes the N-terminally adjacent carbonyl oxygen at 1.63 Å away from the protonated amide carbon atom whereas this distance is 1.53 Å in TS15. The protonated amide bond is elongated to 2.13 Å in TS14, which is 0.40 Å longer than that in TS13. In TS16, the N-terminal amine group is 1.82 Å away from the amide carbonyl carbon and an elongation of the H–K amide bond is up to 2.01 Å.

**Fig. 4:**
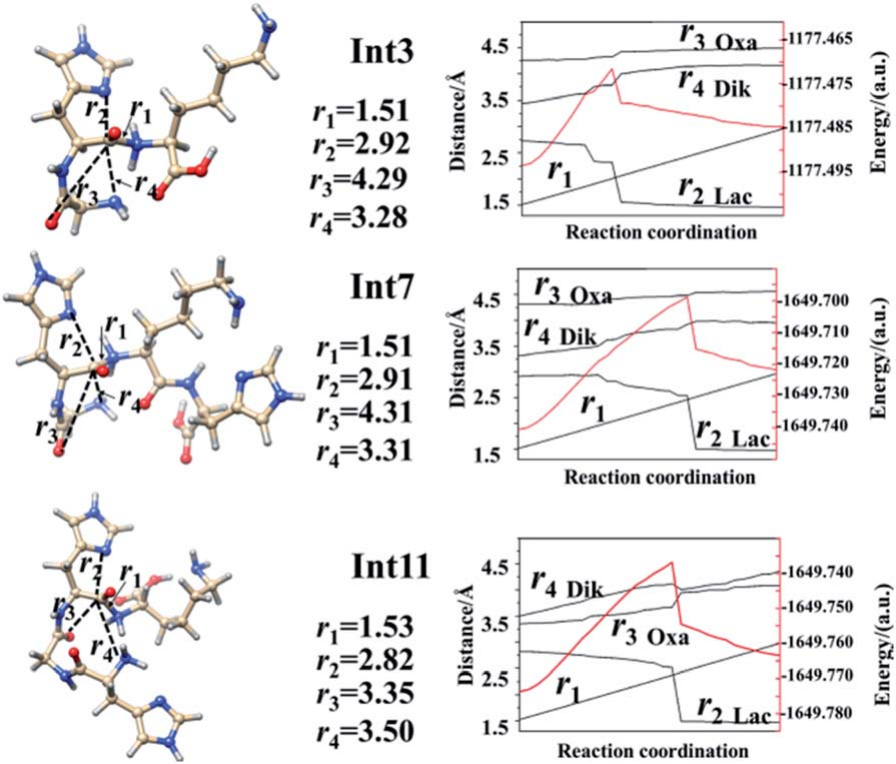
Energy profiles along the scan coordinate of protonated amide bonds (*r*_1_ in images, unit in Å). Left lines are optimized structures while right lines correspond to the energy profiles (unit in a.u.) of the left side structure.

Analyses in terms of relative energy in Fig. 3 and in Schemes 3 and 4 indicate that the Lac pathway is a low energy route for all the protonated peptides, which is confirmed by the energy profile (red line) along scan coordinates of protonated amide bond (*r*_1_) in Fig. 4. The range of the scan coordinates (*r*_1_) increases from about 1.5 to 3.0 Å, where variations of energy profiles and possible reaction pathways (Lac, Oxa and Dik) are investigated. For intermediates Int3, Int7, and Int11, two possible nucleophilic pathways are proposed. Differences of the two pathways lie in that the distance of *r*_4_ decreases firstly in the Dik pathway, where the length of *r*_2_ shortens preferentially in the Lac–Dik pathway. Fig. 4 shows that only distances of *r*_2_ become short quickly along with the cleavage of the protonated bond (*r*_1_), revealing that the nucleophilic attack of the imidazolyl nitrogen of the His takes place preferentially along with the elongation of the H–K amide bond for GHK analogues. This accounts for the lower energy barrier for the Lac pathway.

#### 3.2.4 Analysis of non-covalent interactions

**Fig. 5:**
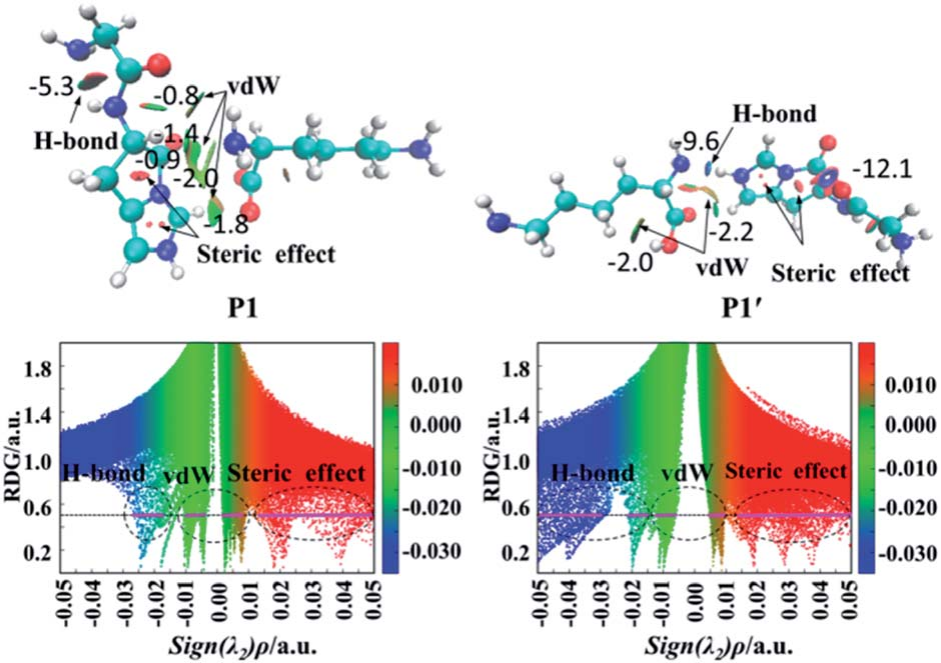
Graphic noncovalent interaction for products (P1 and P1′) obtained from the lactam pathway of the protonated GHK. (Upper panels): Isosurfaces (RDG = 0.5 a.u.). (Lower panels): Scatter maps of RDG *versus* sign(*λ*_2_)*ρ* value, where the RDG isosurface of the horizontal line is 0.5 a.u. The colour scale bar ranges from −0.035 to 0.02.

Non-covalent interaction (NCI) method, also known as the RDG method, is a good approach for investigations of weak interactions.^41^ All graphic NCIs for products are given in Fig. S26–S35 in the ESI,† whereas the graphic NCIs for products (P1 and P1′) obtained from the lactam pathway of the protonated GHK are shown in Fig. 5. In these graphic NCIs, the darkness of the blue colour corresponds to the strength of attractive interactions, where the darkness of the red colour correlates with the strength of the steric interactions. From the colour filled RDG isosurface (RDG = 0.5 a.u.), different types of interaction regions can be identified by simply examining their colour darkness.

Fig. 5 also gives graphically the noncovalent interaction for products P1 and P1′ by RDG isosurfaces and scatter maps. The upper panels correspond to the RDG isosurfaces at RDG = 0.5 a.u. Each colour-filled region is called a slab. For the RDG isosurface of P1, the slab between N-terminal amine group and hydrogen atom of the N-terminally neighboured amide bond shows a deeply green colour, implying that the interaction there is a weak hydrogen bond (H-bond). The lower panels correspond to scatter maps of RDG *versus* the value of sign(*λ*_2_)*ρ*. There are several shapes with the points at their peaks approaching the horizontal axis, which correspond to different types of interactions. A horizontal line was drawn on the graph of RDG = 0.5 a.u, the segments crossing these colour regions corresponded to the points was then used to construct the RDG isosurfaces in the upper panels of this figure. The horizontal line corresponding H-bond segment (lower panels in Fig. 5) is the shortest among the three segments. The slab between carbonyl group of the breaking amide bond and the leaving group is marked by the green region in which a van der Waals (vdW) contact interaction is identified.

Analysis of the scatter maps endows that there are two vdW segments crossing green regions (lower panels in Fig. 5) which are relatively longer than that of an H-bond segment. The vdW region is dominated apparently by the main interactions between the binding of the N-terminal and C-terminal fragments. Besides, regions at the centre of the imidazolyl ring and at the newly formed ring generated by the nucleophilic attack were filled by the red colour, corresponding to steric interactions. The electronic density in these regions is high since the mapped colour is red. The horizontal line crossing the red region is the longest, suggesting strong steric interactions.

The RDG isosurface and scatter map of P1′ are given on right side of Fig. 5. The segment of horizontal line crossing the blue region at RDG = 0.5 a.u is much longer than that of P1, which is also true for other PBDs. The large segment crossing the blue region contributes to a blue H-bond slab between the amine group of truncated C-terminal fragment and the imidazolyl ring of N-terminal fragment.

**Fig. 6:**
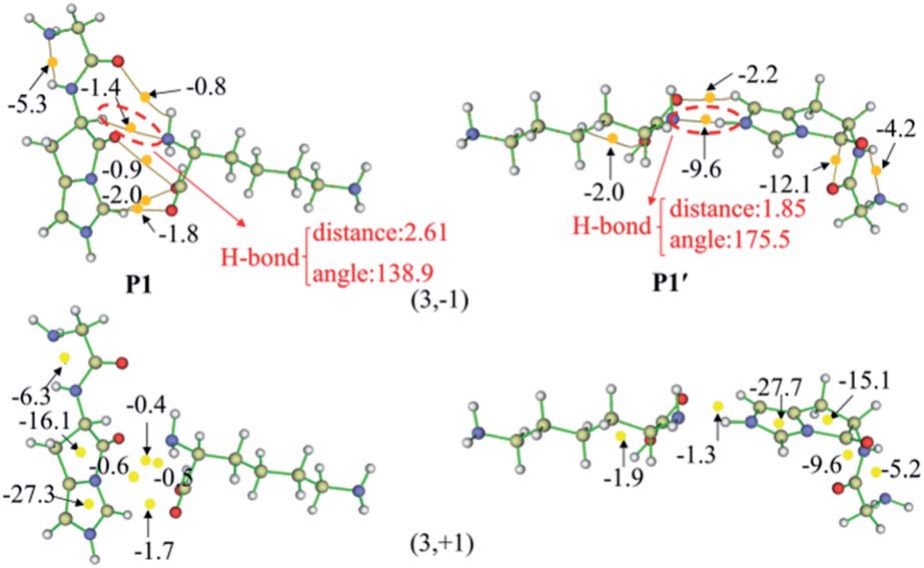
The AIM plots of interaction energies for products P1 and P1′. CPs (3, −1) based on critical points (CPs) with orange spheres (upper panels), CPs (3, +1) with yellow spheres (lower panels), and the bond paths connecting (3, −1) with brown lines.

The AIM plots of interacting energies for products P1 and P1′ based on critical points (CPs) are shown in Fig. 6, while similar plots for other products are given in Fig. S36–S46 in the ESI.† For electron density analysis, the bond critical point (BCP) (3, −1) coloured in the orange sphere reflects the atomic interactions, and it has higher electron density if the calculated *E*_HB_ values are more negative. For P1, no *E*_HB_ values are more than 2.0 kcal mol^−1^ between the N-terminal and C-terminal fragments, even the sum of the *E*_HB_ values is only about −6.9 kcal mol^−1^. Thus, the truncated C-terminal fragment interacts with the protonated N-terminal fragment weakly. These small values are consistent with the green slabs between carbonyl group of the breaking amide bond and the truncated department shown in Fig. 5. Hence, the vdW interactions are confirmed by these small *E*_HB_ values. Structural analyses indicate that the truncated C-terminus only forms a weak H-bond (C–H⋯N) with a distance of 2.61 Å (upper panels of Fig. 6). As to P1′, the recombination of the hydrogen bonding model generates strong interactions by hydrogen bond with the bond distance of 1.85 Å (the upper panels of Fig. 6). The amine group of the truncated C-terminal fragment can form a strong hydrogen bond with the positively charged imidazolyl ring of the His, the calculated *E*_HB_ value is up to −9.6 kcal mol^−1^. The largely negative value of *E*_HB_ certainly contributes to the blue H-bond slab in the RDG isosurface (Fig. 5).

For electron density analysis, CP (3, +1) is often named as ring critical point (RCP), generally displaying the steric interactions. Interaction energies based on CPs (3, +1) for products P1 and P1′ are also shown in Fig. 6 with yellow sphere. Those energies obtained from the CPs (3, +1) between carbonyl group of the breaking amide bond and the truncated department are all small. On the other hand, the interaction energies generated from the CPs (3, +1) in the centre of ring are much larger. For P1 and P1′, each contains two rings which are the imidazolyl ring and the newly formed ring generated by the nucleophilic attack of the His side chain. The estimated interaction energy in the centre of the imidazolyl ring is almost constant (−27.3 kcal mol^−1^ for P1*versus* −27.7 kcal mol^−1^ for P1′), where the energy in the centre of newly formed five-membered ring changes slightly (−16.1 kcal mol^−1^ for P1*versus* −15.1 kcal mol^−1^ for P1′).

**Fig. 7:**
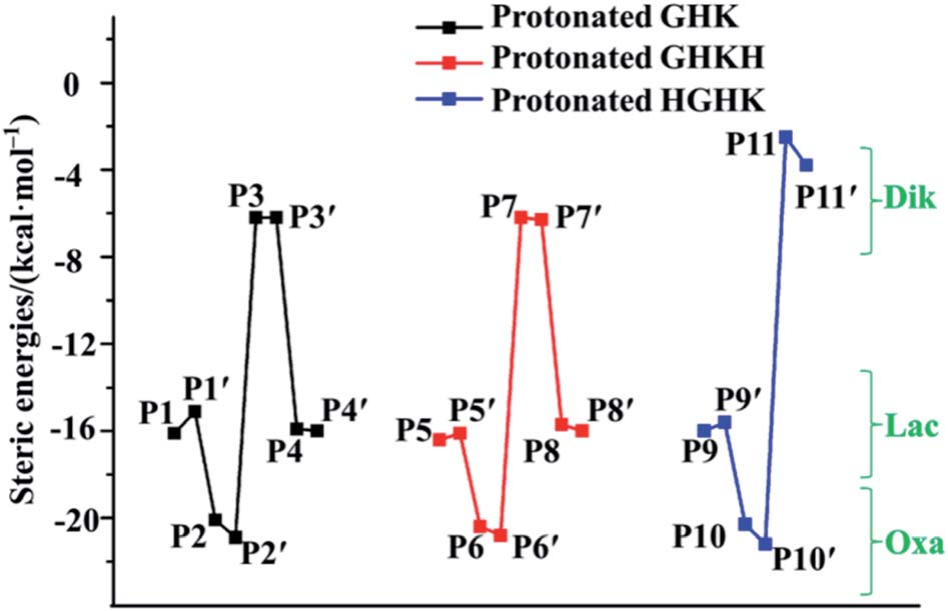
Relations of steric interaction energies obtained on CPs (3, +1); unit in kcal mol^−1^.

Examination of readiness of formation of the five-membered rings in the Lac and Oxa pathways and of six/nine-membered rings in the Dik pathway is of critical importance. Consequently, the relations of interaction energies in the centre of formed rings are depicted in Fig. 7, demonstrating that all estimated steric interaction energies in the Oxa pathway (P2 and P2′; P6 and P6′; P10 and P10′) are the largest among all the pathways. Those derived in the Dik pathway (P3 and P3′; P7 and P7′; P11 and P11′) are the smallest. Indeed, our results support the six-membered ring structure in the cases of GHK and GHKH and the nine-membered ring structure in the case of HGHK in the Dik pathway.^12,13,22^ The statistic values of attractive and steric interaction energies in the N-terminal and C-terminally truncated fragments are summarized in Table 2. An overall analysis unveils that the steric interactions are much larger than attractive interactions. Furthermore, the total attractive interactions become much big when the PBD is formed, *i.e.*, the attractive interaction energy in P1 is −12.4 kcal mol^−1^, while it adds up to −25.9 kcal mol^−1^ in P1′. Clearly, the formation of the PBD enhances the stability.

**Table 2:** Statistic values of the estimated attractive and steric interaction between the N-terminal fragments and C-terminally truncated fragments

Products	Total of attractive interaction/(kcal mol^−1^)	Total of steric effect/(kcal mol^−1^)
P1	−12.4	−52.9
P1′	−25.9	−60.8
P2	−14.2	−62.3
P2′	−21.2	−53.6
P3	−26.3	−37.5
P3′	−48.4	−43.5
P4	−16.7	−48.3
P4′	−21.3	−50.9
P5	−27.8	−78.8
P5′	−51.6	−90.5
P6	−11.4	−70.8
P6′	−48.9	−87.5
P7	−36.3	−72.6
P7′	−59.8	−72.1
P8	−18.8	−79.9
P8′	−23.6	−83.3
P9	−34.5	−84.3
P9′	−30.1	−85.2
P10	−20.6	−88.3
P10′	−26.8	−90.4
P11	−38.2	−75.9
P11′	−46.7	−72.8

## 4. Conclusions

Fragmenting mechanisms of the singly protonated GHK and its synthetic analogues GHKH and HGHK have been investigated by electrospray ionization tandem mass spectrometry, whereas the H–K amide bond breaking mechanisms are further investigated by theoretical methods. Fragmentations at the H–K amide bond of the protonated GHK and GHKH are the dominant cleavages observed experimentally. In the case of HGHK, the N-terminal His hampers significantly the breaking of H–K amide bond due to a high energy barrier. Theoretical calculations indicate that the His effect plays an important role for the H–K amide bond fragmentation. The energy barriers of the lactam pathway are lower than those of the classical Oxa and the Dik pathways. The recombination processes of the intermediate products being transferred to the PBDs are favoured thermodynamically since these transformations are all exergonic. We further demonstrated that formation of PBDs is definitely feasible which can reduce the energy barriers significantly for all the reaction pathways. A comparison of the RDG isosurface with the scatter map suggests that hydrogen bonding contributes largely to the stabilities of PBDs. An AIM analysis of the interaction energies in the newly formed ring at critical points (3, +1) indicate that a six-membered and a nine-membered ring structures generated in the Dik pathway is favoured dynamically when compared with products generated from the Lac or the Oxa pathways. This is the first feasibility investigation for the formation of different rings generated in the Lac, Oxa or Dik pathways from a dynamic perspective.

## Conflicts of interest

The authors declare no conflict of interest.

## Supplementary Material

RA-011-D0RA07600G-s001
