# Supplementary material for: Mass spectral and theoretical investigations of the transient proton-bound dimers on the cleavage processes of the peptide GHK and its analogues
Source: RSC Adv. 2021 Jan 20;11(7):4077–86. doi: 10.1039/d0ra07600g (PMC8694339; doi:10.1039/d0ra07600g)
Supplement: RA-011-D0RA07600G-s001 [file RA-011-D0RA07600G-s001.pdf]

Electronic Supplementary Material (ESI) for RSC Advances.
This journal is © The Royal Society of Chemistry 2021

Electronic Supplementary Information for:

Mass spectral and theoretical investigations of the transient proton-bound dimers on the cleavage processes of the peptide GHK and its analogues

Jinhu Wang,[a,*] Cheng Wang,[b] Han Zhang,[a] Yang Liu,[a] Tiesheng Shi [a]

[a] College of Chemistry, Chemical Engineering and Materials Science, Zaozhuang University, Zaozhuang 277160, Shandong Provoince, P. R. China

[b] Department of Traditional Chinese Medicine, Zaozhuang Municipal Hospital, Zaozhuang 277102, Shandong Province, P. R. China

Electronic Supplementary Information includes 3 supporting Tables (Tables S1-S3), and 46 supporting figures (Figures S1-S46).

Table S1 Energies of protonated GHK conformations at the B3LYP/def2-TZVP level of theory.

Structures	E_{total}/a.u	$E_{total+ZPE}$/a.u	Relative energies/(kcal/mol)
**R1**	-1177.543282	-1177.106026	0.0
**Int1**	-1177.509664	-1177.07489	19.5
**TS1**	-1177.492973	-1177.058901	29.6
**P1**	-1177.501736	-1177.067298	24.3
**P1′**	-1177.513063	-1177.079776	16.5
**TS2**	-1177.474819	-1177.040602	41.1
**P2**	-1177.478704	-1177.043709	39.1
**P2′**	-1177.500339	-1177.067961	23.9
**TS3**	-1177.487533	-1177.057512	30.4
**Int2**	-1177.504783	-1177.070277	22.4
**R1′**	-1177.529514	-1177.093813	7.7
**Int3**	-1177.489535	-1177.055767	31.5
**TS4**	-1177.476046	-1177.042644	39.8
**P3**	-1177.492101	-1177.05871	29.7
**P3′**	-1177.517278	-1177.083513	14.1
**TS5**	-1177.479971	-1177.047497	36.7
**P4**	-1177.485672	-1177.051956	33.9
**P4′**	-1177.502203	-1177.06829	23.7
**TS6**	-1177.468762	-1177.035111	44.5
**Int4**	-1177.471852	-1177.037758	42.8

Table S2 Energies of protonated GHKH conformations at the B3LYP/def2-TZVP level of theory.

Structures	E_{total}/a.u	$E_{total+ZPE}$/a.u	Relative energies/(kcal/mol)
**R2**	-1649.810317	-1649.232007	0.0
**Int5**	-1649.772145	-1649.196106	22.5
**TS7**	-1649.757793	-1649.182764	30.9
**P5**	-1649.773791	-1649.198452	21.1
**P5′**	-1649.782941	-1649.206143	16.2
**TS8**	-1649.733631	-1649.158777	46.0
**P6**	-1649.744045	-1649.168144	40.1
**P6′**	-1649.755858	-1649.181372	31.8
**TS9**	-1649.743813	-1649.17237	37.4
**Int6**	-1649.762956	-1649.189416	26.7
**R2′**	-1649.796574	-1649.219677	7.7
**Int7**	-1649.74056	-1649.16442	42.4
**TS10**	-1649.727285	-1649.150917	50.9
**P7**	-1649.746883	-1649.173618	36.6
**P7′**	-1649.77234	-1649.196067	22.6
**TS11**	-1649.707924	-1649.134698	61.1
**P8**	-1649.722718	-1649.149627	51.7
**P8′**	-1649.754768	-1649.178638	33.5
**TS12**	-1649.718328	-1649.143374	55.6
**Int8**	-1649.721178	-1649.144912	54.7

Table S3 Energies of protonated HGHK conformations at the B3LYP/def2-TZVP Level of theory.

Structures	E_{total}/a.u	$E_{total+ZPE}$/a.u	Relative energies/(kcal/mol)
**R3**	-1649.797908	-1649.219933	0.0
**Int9**	-1649.756654	-1649.179991	25.1
**TS13**	-1649.737749	-1649.161985	36.4
**P9**	-1649.743515	-1649.166679	33.4
**P9′**	-1649.777421	-1649.200891	11.9
**TS14**	-1649.715587	-1649.140660	49.7
**P10**	-1649.740468	-1649.164756	34.6
**P10′**	-1649.753320	-1649.179172	25.6
**TS15**	-1649.739412	-1649.167064	33.2
**Int10**	-1649.753577	-1649.177152	26.8
**Int11**	-1649.773648	-1649.194413	16.0
**TS16**	-1649.714090	-1649.137314	51.8
**P11**	-1649.719945	-1649.143015	48.3
**P11′**	-1649.761251	-1649.187802	20.2

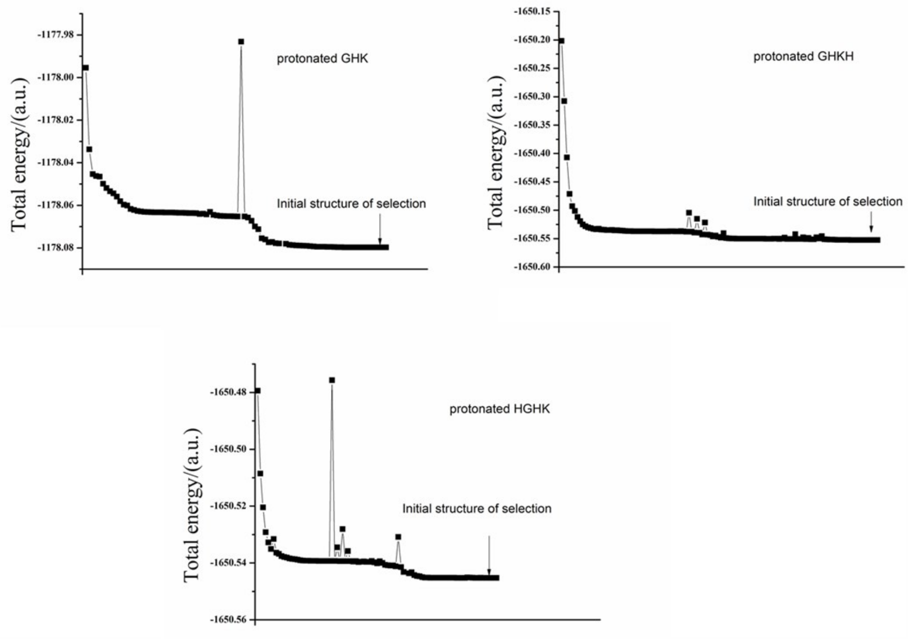

Figure S1. Single point energy calculations of many structures sampled from the MD trajectory at at the B3LYP/def-TZVP level using Turbomole.

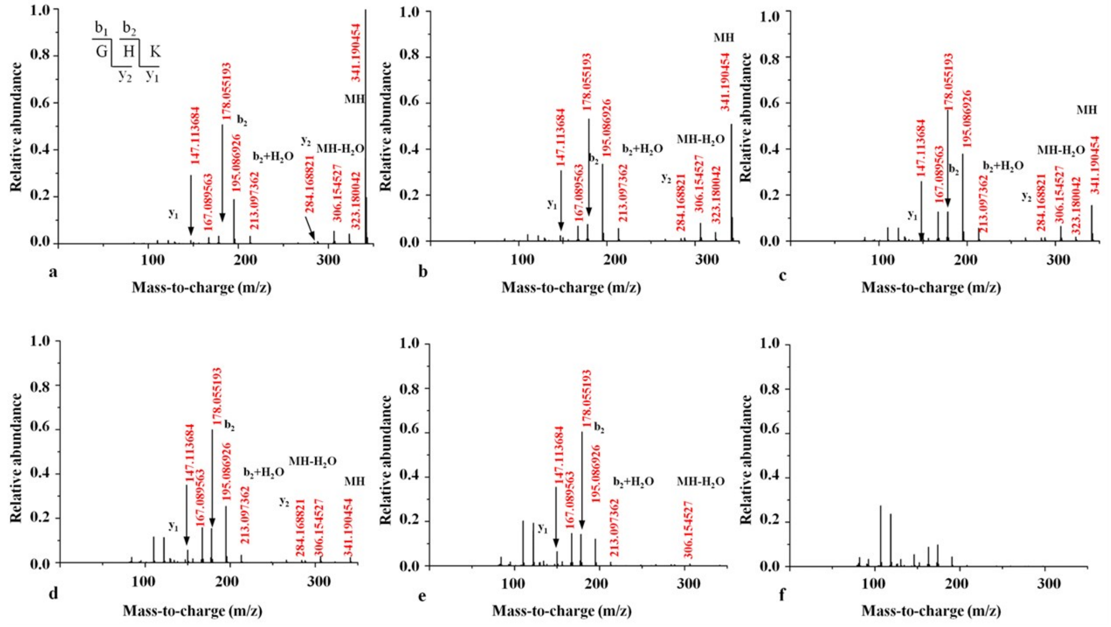

Figure S2. MS/MS spectra for GHK with different collision energies: (a) 5.0 eV; (b) 10.0 eV; (c) 15.0 eV; (d) 20.0 eV; (e) 25.0 eV and (f) 30.0 eV.

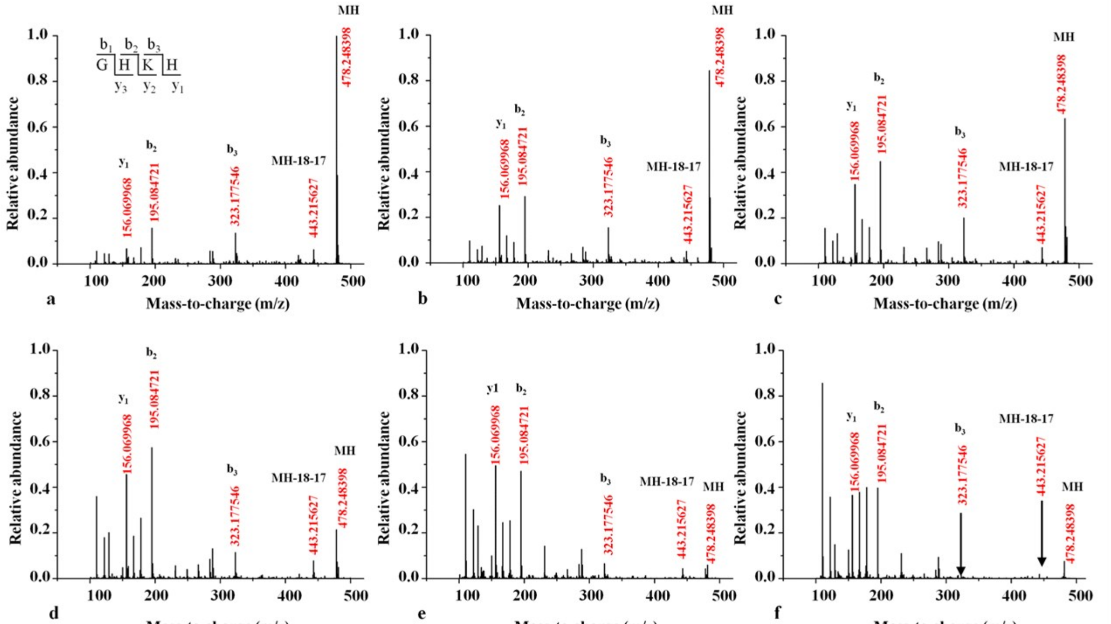

Figure S3. MS/MS spectra for GHKH with different collision energies: (a) 5.0 eV; (b) 10.0 eV; (c) 15.0 eV; (d) 20.0 eV; (e) 25.0 eV and (f) 30.0 eV.

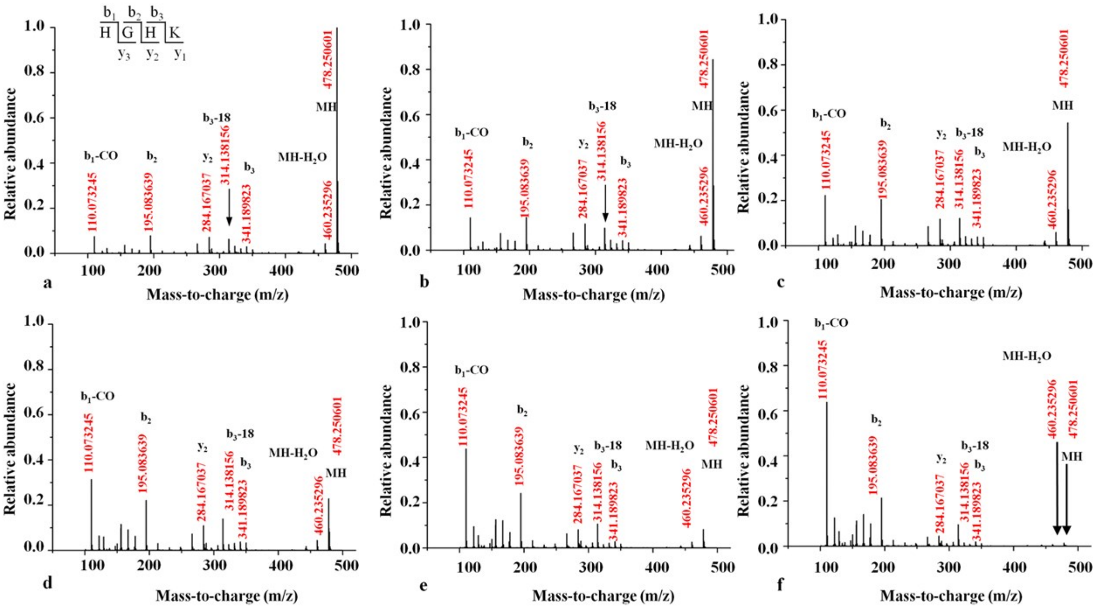

Figure S4. MS/MS spectra for HGHK with different collision energies: (a) 5.0 eV; (b) 10.0 eV; (c) 15.0 eV; (d) 20.0 eV; (e) 25.0 eV and (f) 30.0 eV.

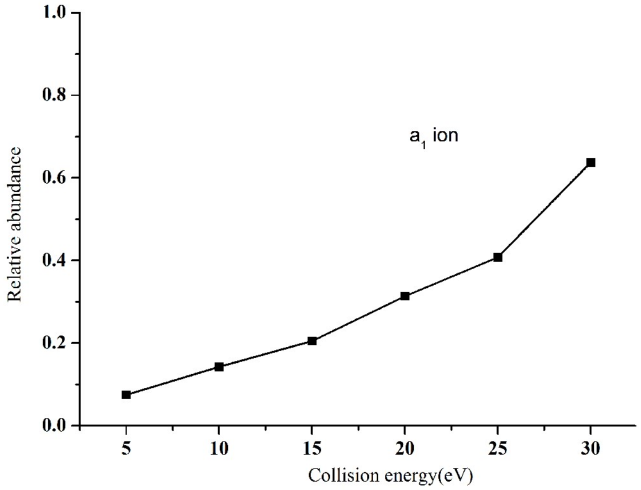

Figure S5. Relative abundances for the H–G amide bond fragmentations for the singly protonated HGHK peptidyl ion.

Lac pathway

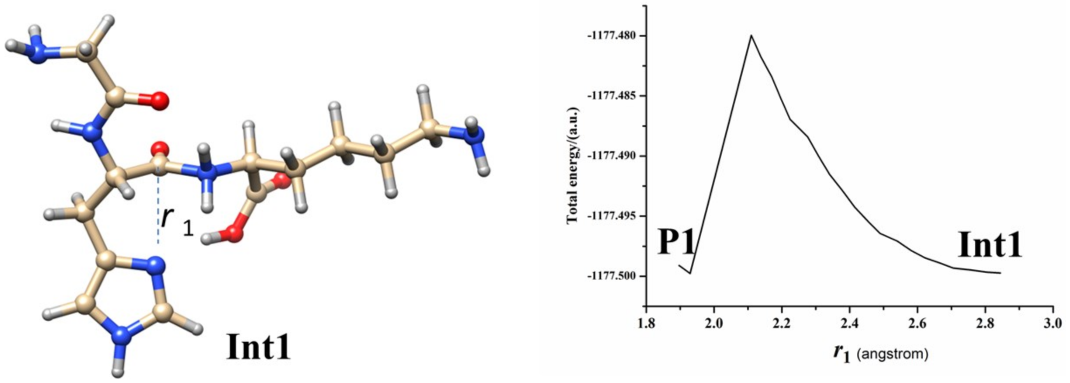

Figure S6. Lac pathway of the singly protonated GHK scanned by r_1 distance as the reaction coordinates.

Oxa-Lac pathway

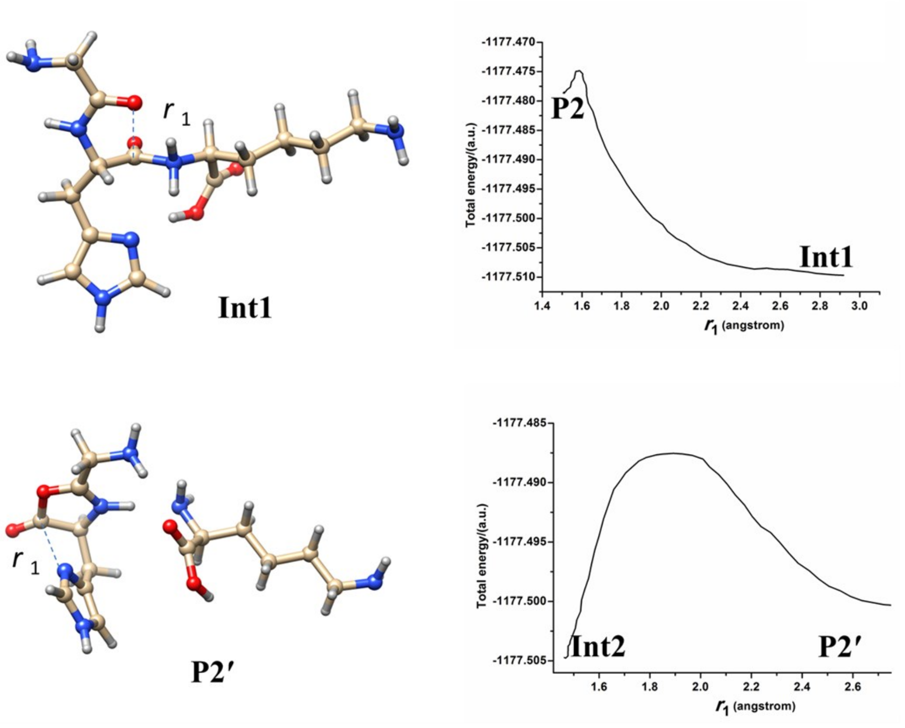

Figure S7. Oxa-Lac pathway of the singly protonated GHK scanned by r_1 distance as the reaction coordinates.

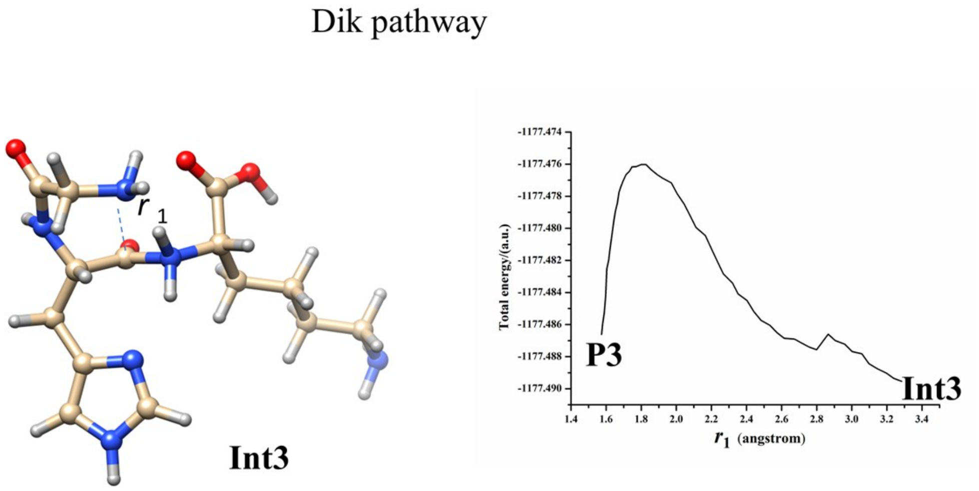

Figure S8. Dik pathway of the singly protonated GHK scanned by r_1 distance as the reaction coordinates.

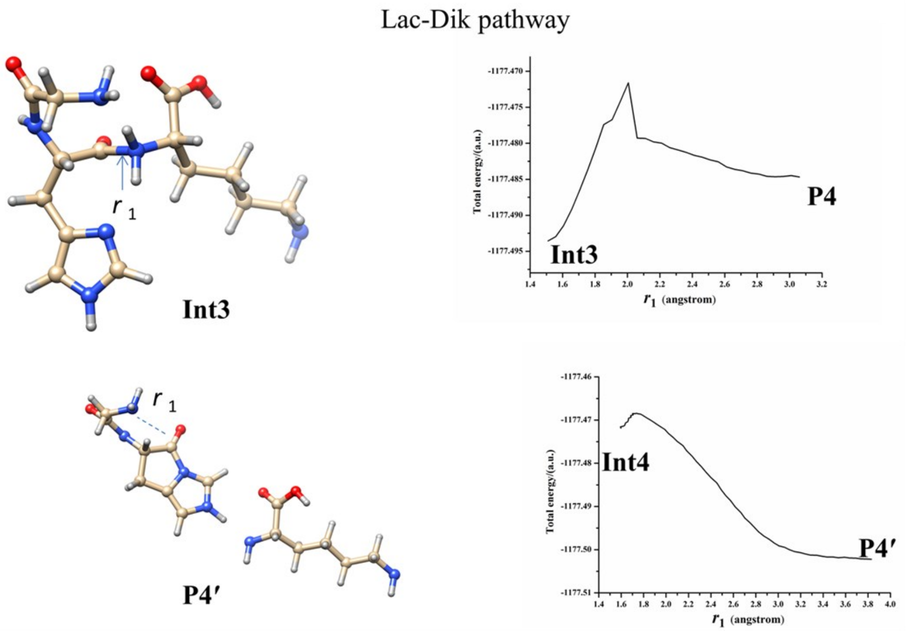

Figure S9. Lac-Dik pathway of the singly protonated GHK scanned by r_1 distance as the reaction coordinates.

Lac pathway

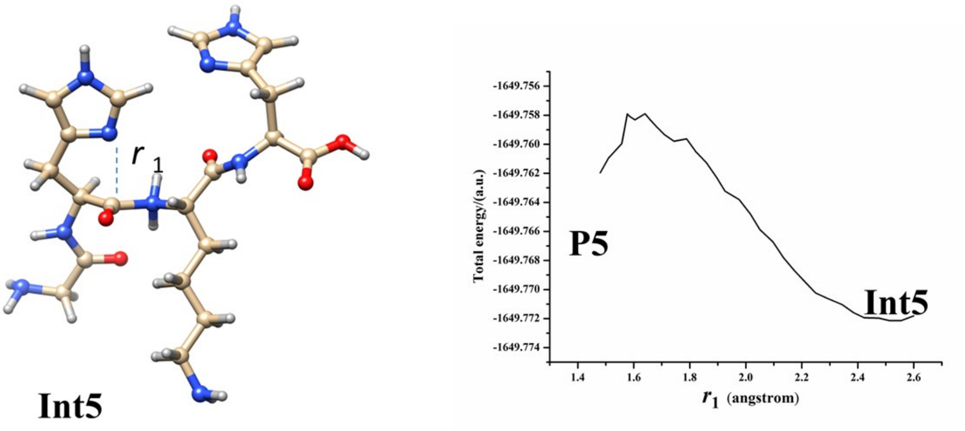

Figure S10. Lac pathway of the singly protonated GHKH scanned by r_1 distance as the reaction coordinates.

Oxa-Lac pathway

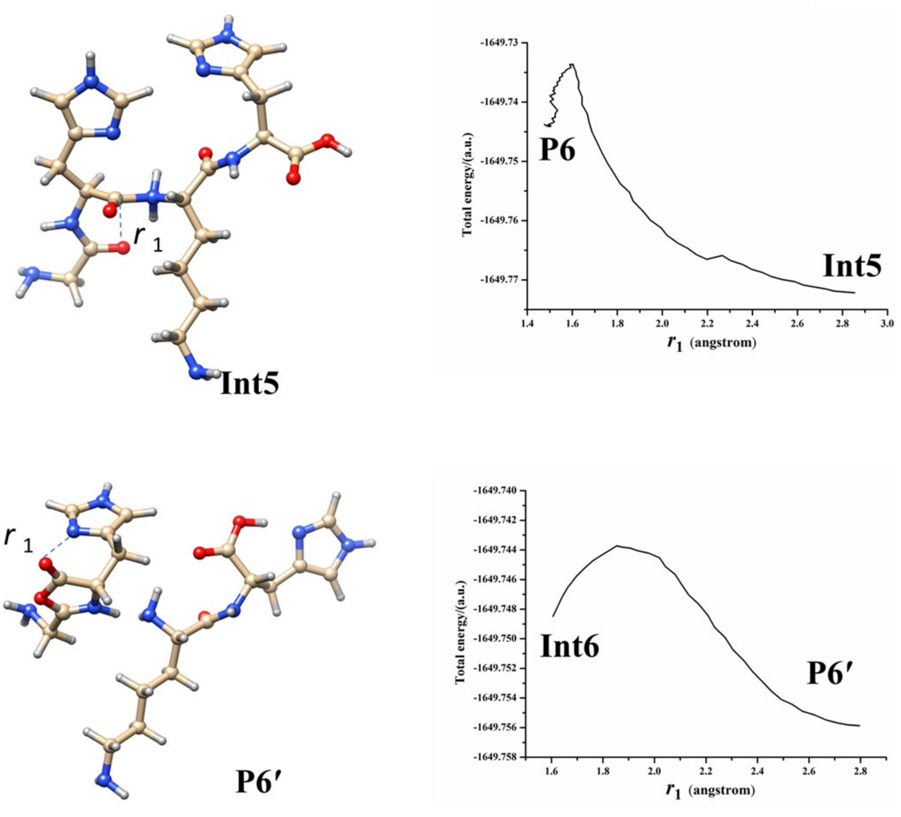

Figure S11. Oxa-Lac pathway of the singly protonated GHKH scanned by r_1 distance as the reaction coordinates.

Dik pathway

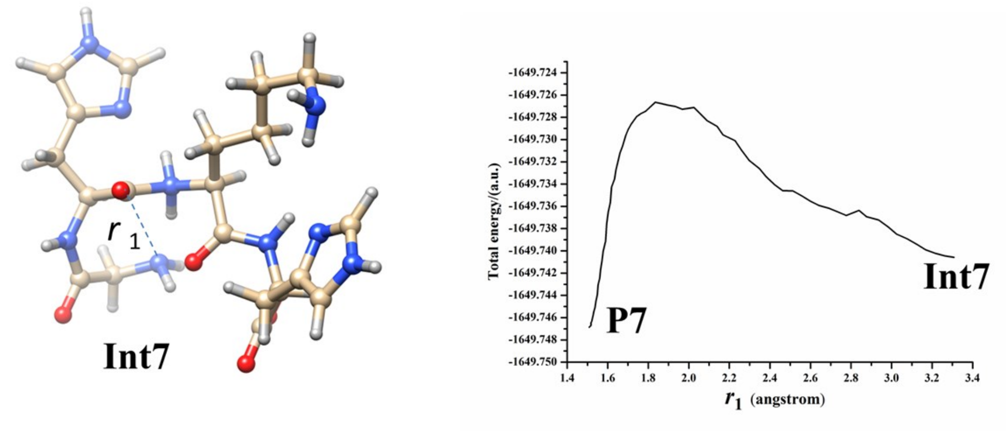

Figure S12. Dik pathway of the singly protonated GHKH scanned by r_1 distance as the reaction coordinates.

Lac-Dik pathway

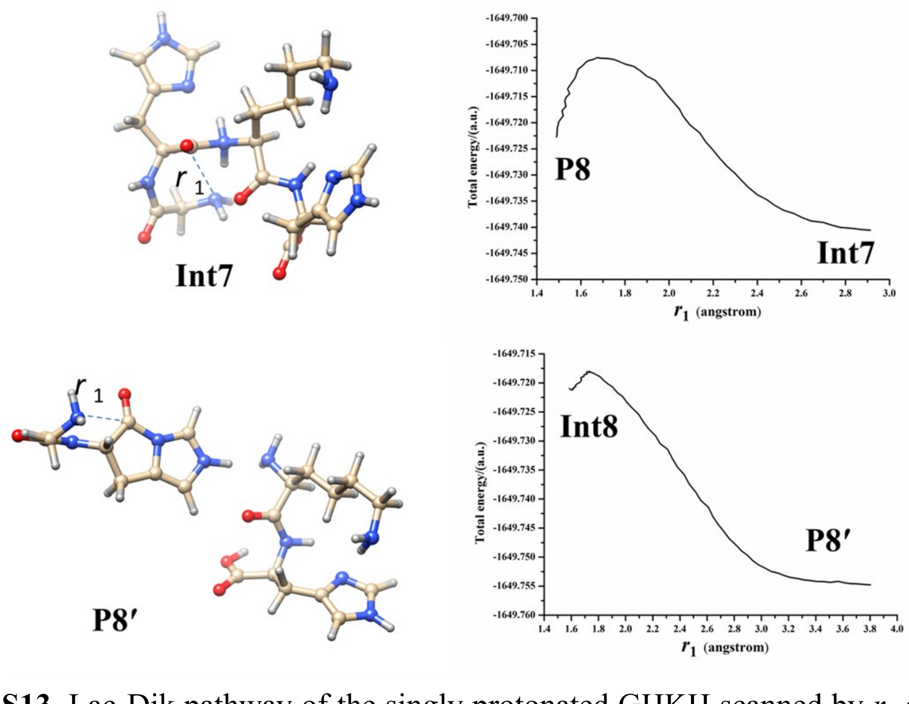

Figure S13. Lac-Dik pathway of the singly protonated GHKH scanned by r_1 distance as the reaction coordinates.

Lac pathway

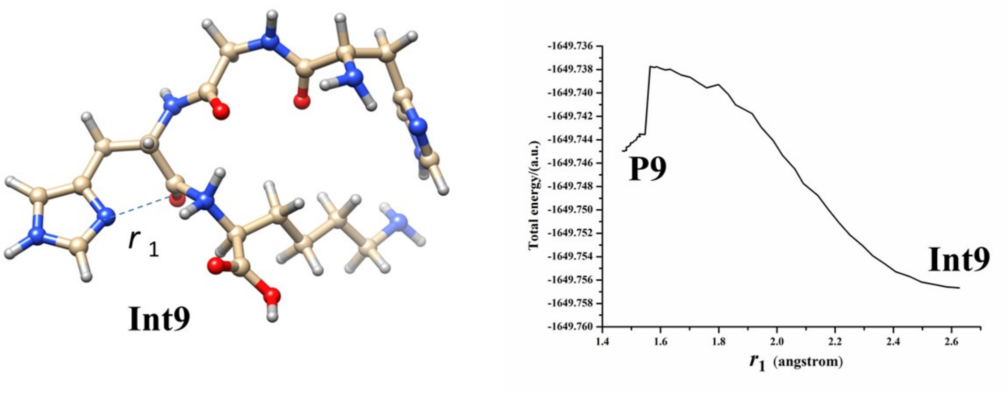

Figure S14. Lac pathway of the singly protonated HGHK scanned by r_1 distance as the reaction coordinates.

Oxa-Lac pathway

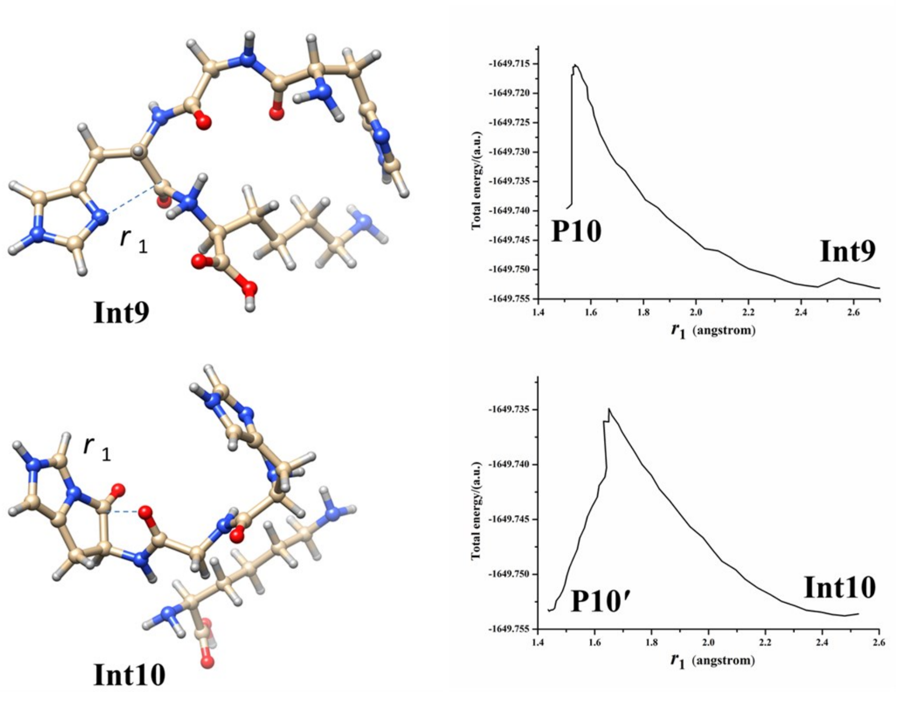

Figure S15. Oxa-Lac pathway of the singly protonated HGHK scanned by r_1 distance as the reaction coordinates.

Dik pathway

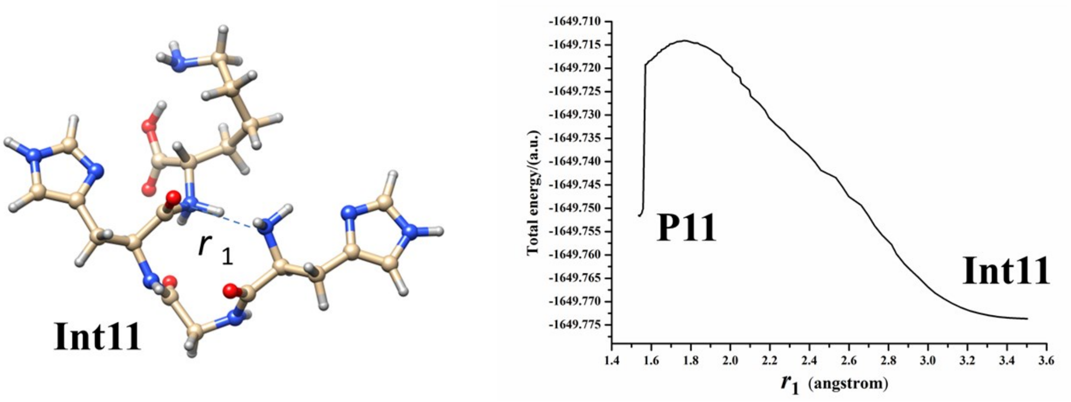

Figure S16. Dik pathway of the singly protonated HGHK scanned by r_1 distance as the reaction coordinates

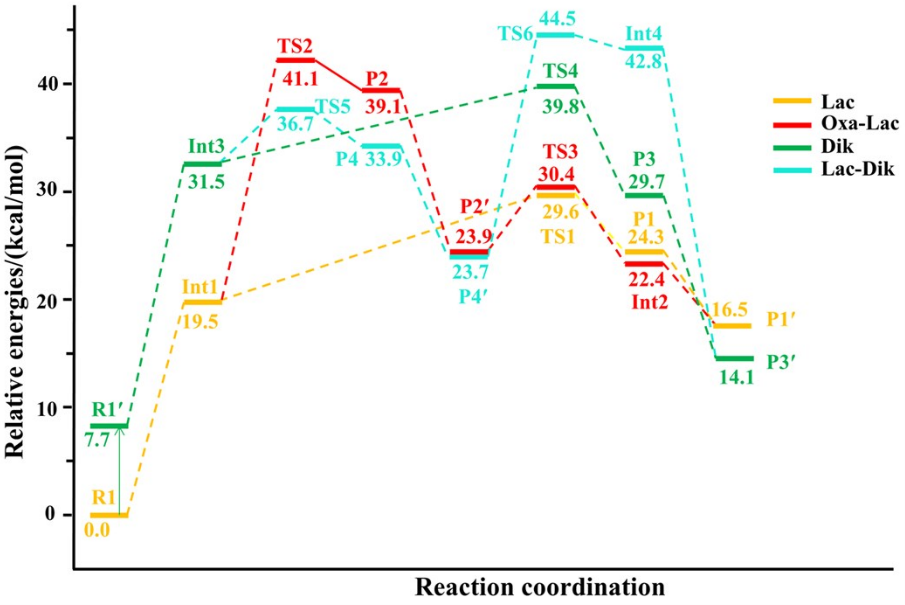

Figure S17. Energy profile for the H-K amide bond cleavage of protonated GHK.

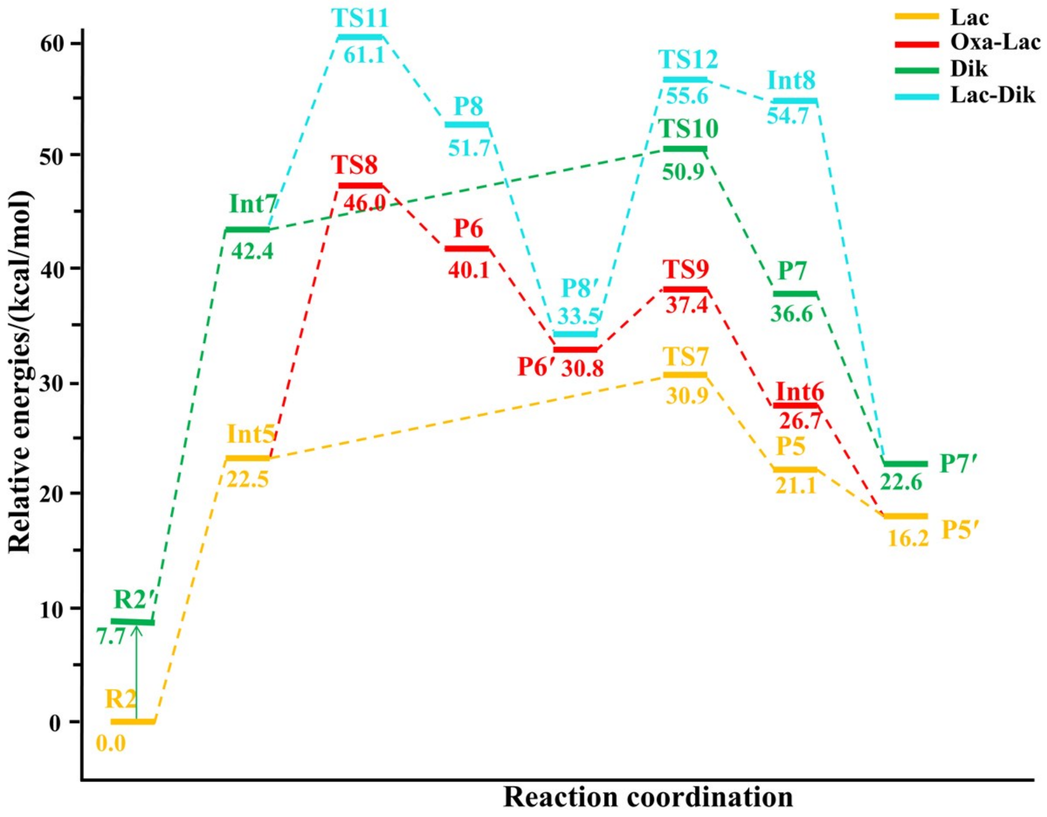

Figure S18. Energy profile for protonated GHKH.

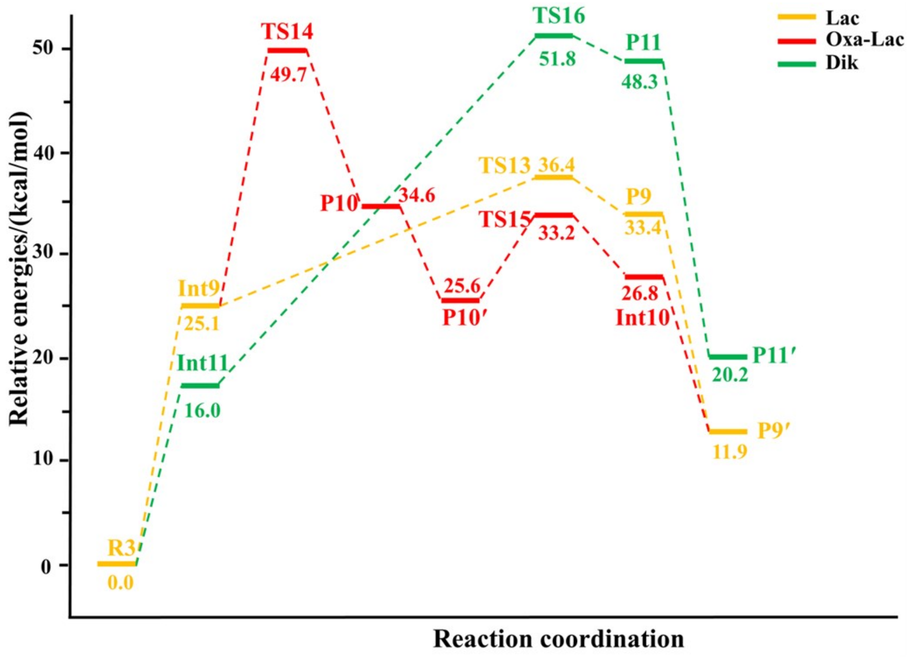

Figure S19. Energy profile for protonated HGHK.

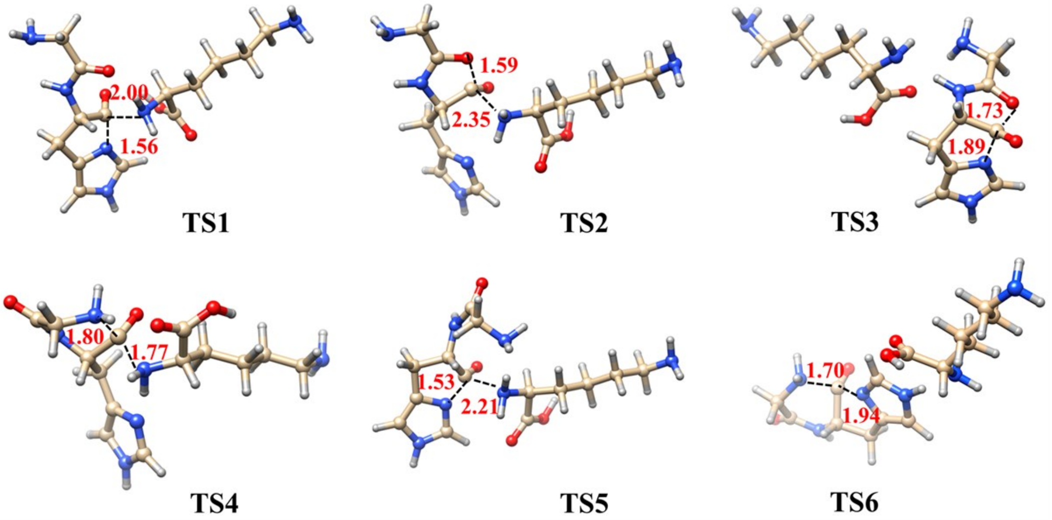

Figure S20. Optimized transition state structures obtained from the H-K amide bond cleavage of protonated GHK.

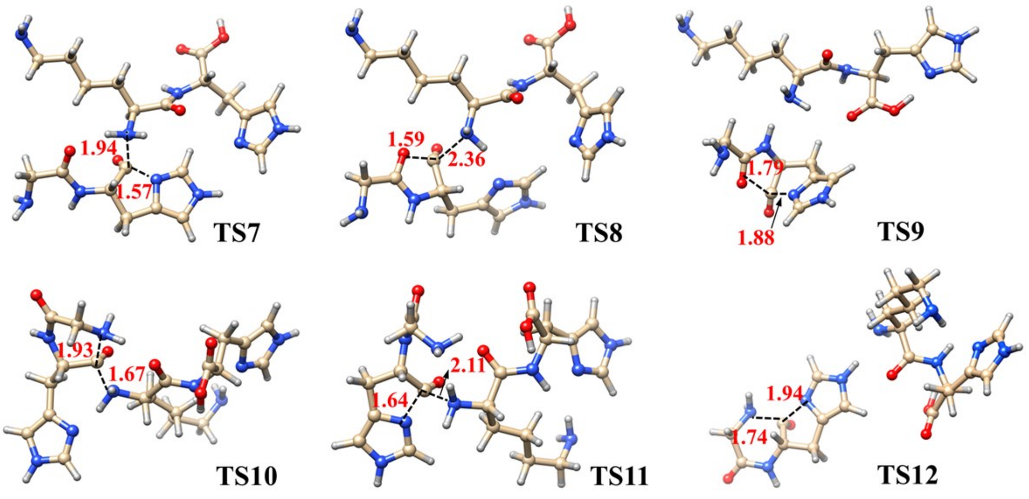

Figure S21. Optimized transition state structures obtained from the H-K amide bond cleavage of protonated GHKH.

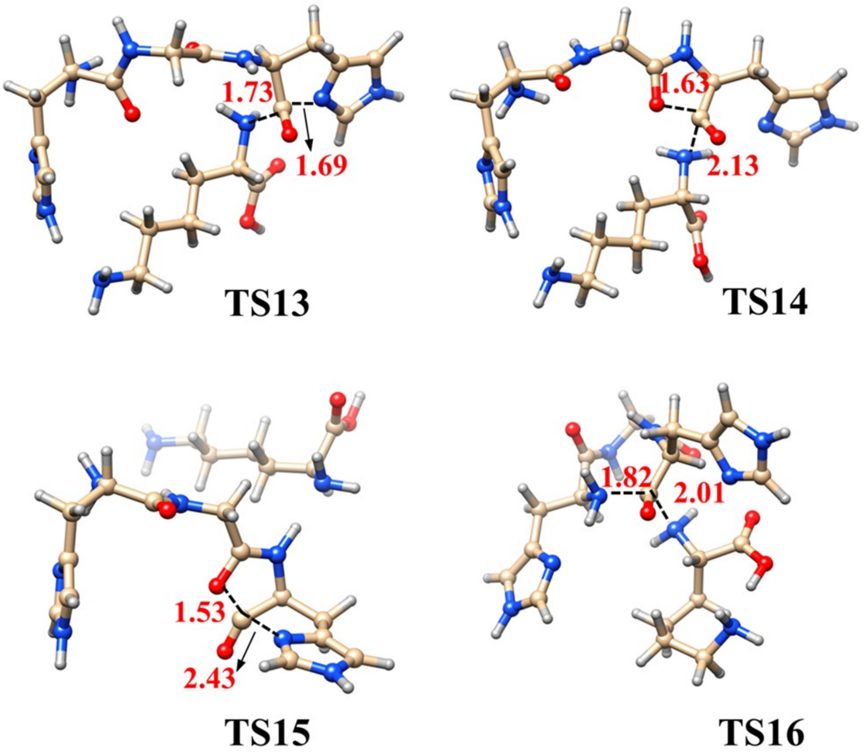

Figure S22. Optimized transition state structures obtained from the H-K amide bond cleavage of protonated HGHK.

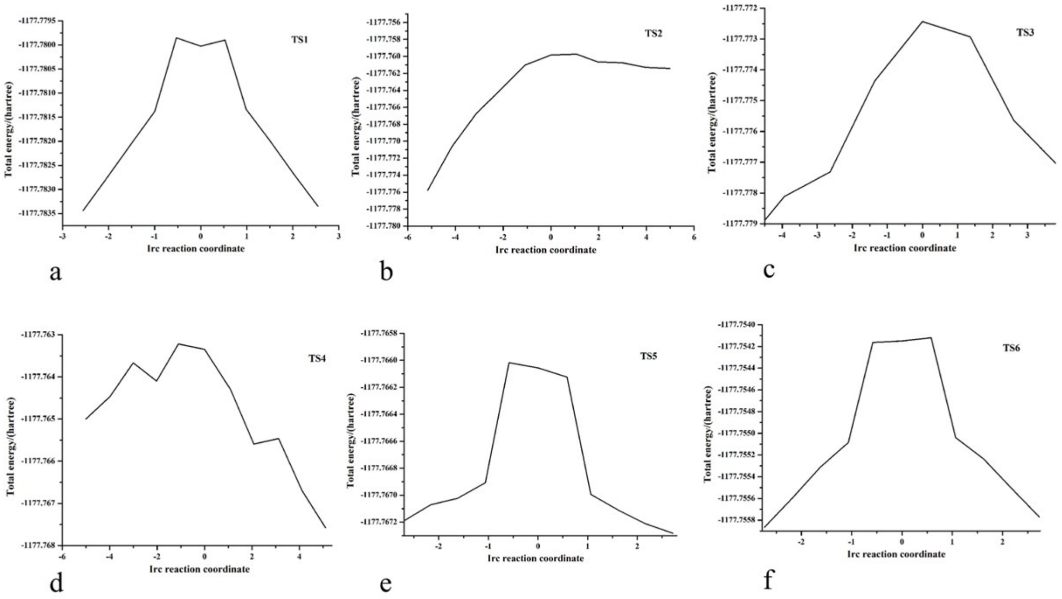

Figure S23. Total energy along irc reaction coordinate for different TSs for protonated GHK.

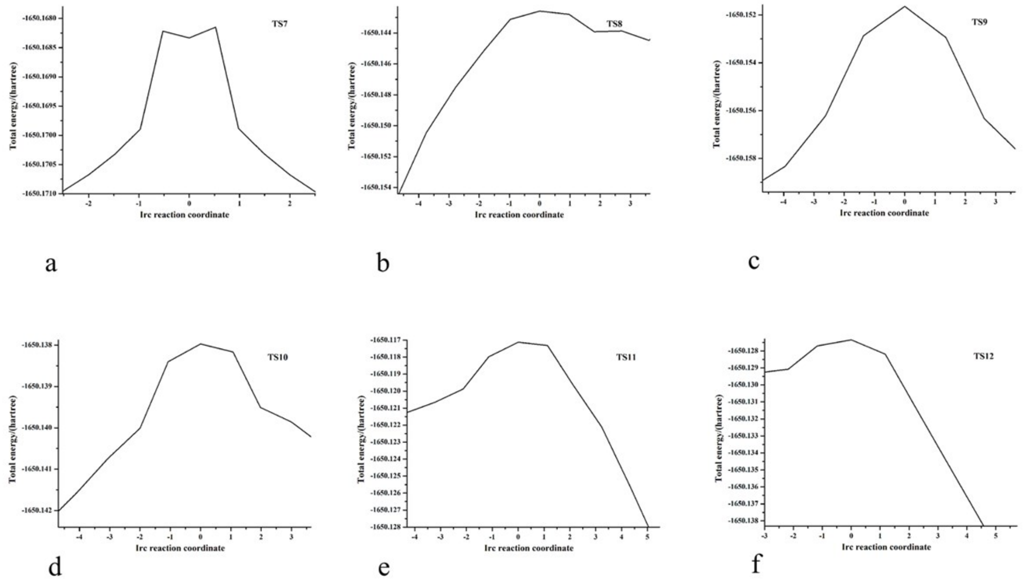

Figure S24. Total energy along irc reaction coordinate for different TSs for protonated GHKH.

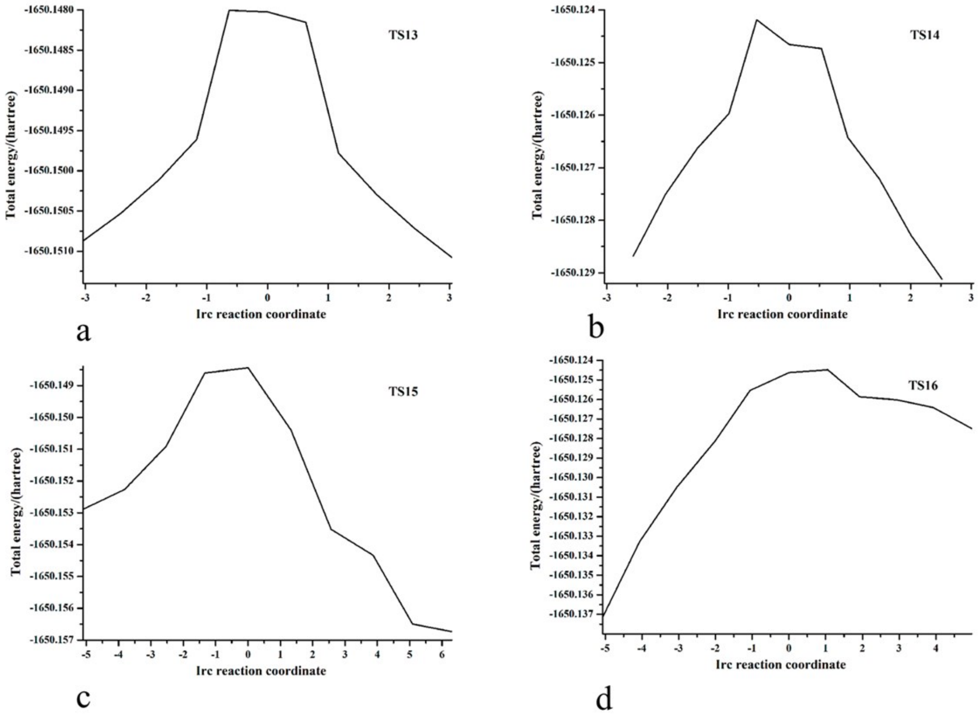

Figure S25. Total energy along irc reaction coordinate for different TSs for protonated HGHK.

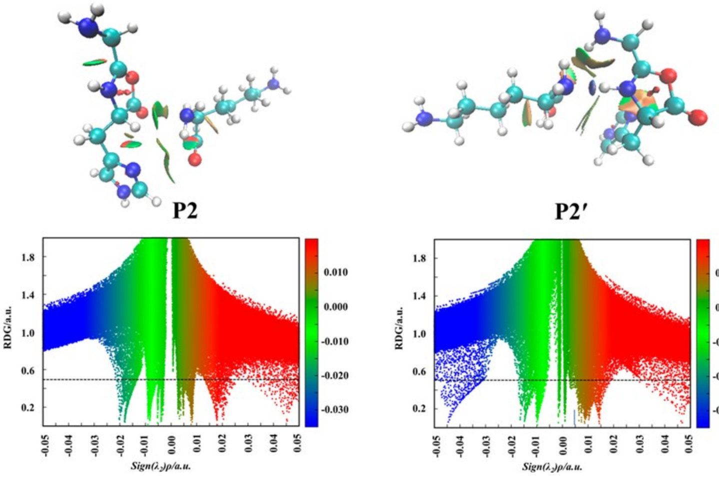

Figure S26. Graphic noncovalent interaction for products (P2 and P2′) obtained from the lactam pathway of protonated GHK. The upper corresponds to RDG isosurfaces (RDG = 0.5 a.u.); the lower corresponds to scatter maps of RDG vs sign(λ_2)ρ value, where the RDG isosurface of the horizontal line is 0.5 a.u. The color scale bar ranges from -0.035 to 0.02.

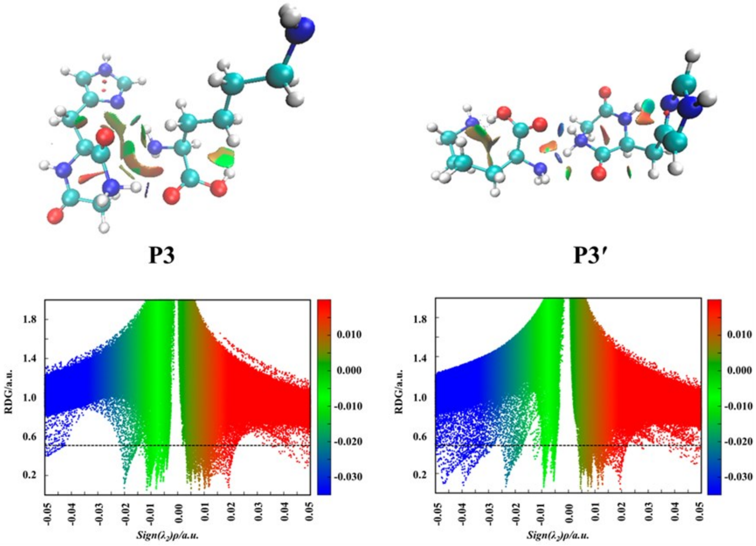

Figure S27. Graphic noncovalent interaction for products (**P3** and **P3′**) obtained from the lactam pathway of protonated GHK. The upper corresponds to RDG isosurfaces (RDG = 0.5 a.u.); the lower corresponds to scatter maps of RDG vs sign(λ_2)ρ value, where the RDG isosurface of the horizontal line is 0.5 a.u. The color scale bar ranges from -0.035 to 0.02.

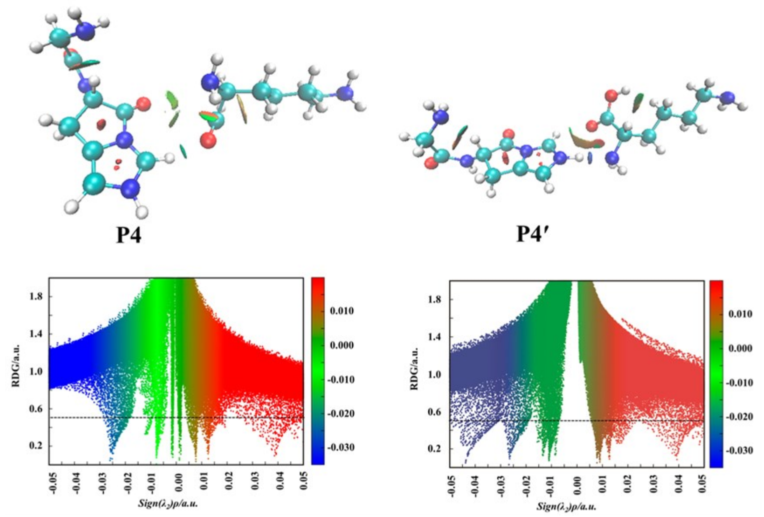

Figure S28. Graphic noncovalent interaction for products (**P4** and **P4′**) obtained from the lactam pathway of protonated GHK. The upper corresponds to RDG isosurfaces (RDG = 0.5 a.u.); the lower corresponds to scatter maps of RDG vs sign(λ_2)ρ value, where the RDG isosurface of the horizontal line is 0.5 a.u. The color scale bar ranges from -0.035 to 0.02.

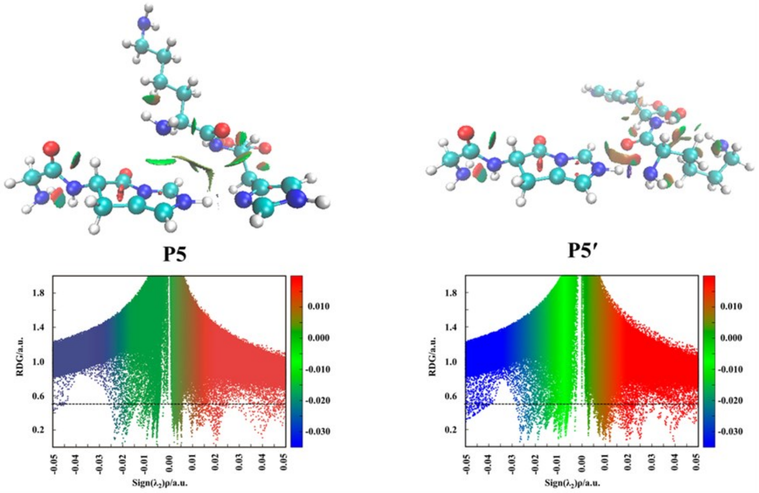

Figure S29. Graphic noncovalent interaction for products (**P5** and **P5′**) obtained from the lactam pathway of protonated GHKH. The upper corresponds to RDG isosurfaces (RDG = 0.5 a.u.); the lower corresponds to scatter maps of RDG vs sign(λ_2)ρ value, where the RDG isosurface of the horizontal line is 0.5 a.u. The color scale bar ranges from -0.035 to 0.02.

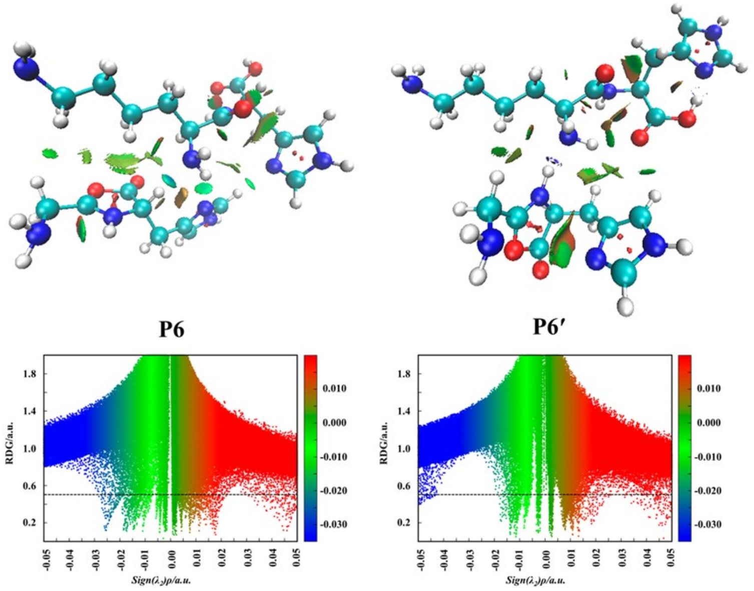

Figure S30. Graphic noncovalent interaction for products (**P6** and **P6′**) obtained from the lactam pathway of protonated GHKH. The upper corresponds to RDG isosurfaces (RDG = 0.5 a.u.); the lower corresponds to scatter maps of RDG vs sign(λ_2)ρ value, where the RDG isosurface of the horizontal line is 0.5 a.u. The color scale bar ranges from -0.035 to 0.02.

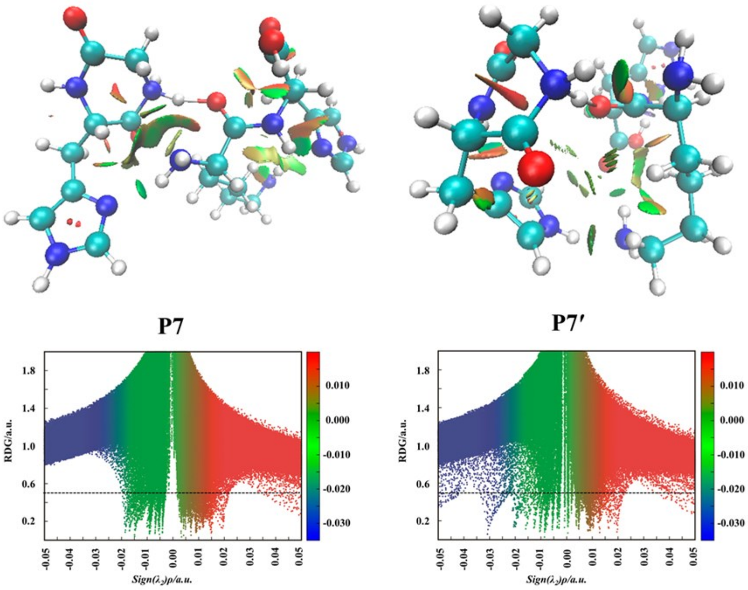

Figure S31. Graphic noncovalent interaction for products (**P7** and **P7′**) obtained from the lactam pathway of protonated GHKH. The upper corresponds to RDG isosurfaces (RDG = 0.5 a.u.); the lower corresponds to scatter maps of RDG vs sign(λ_2)ρ value, where the RDG isosurface of the horizontal line is 0.5 a.u. The color scale bar ranges from -0.035 to 0.02.

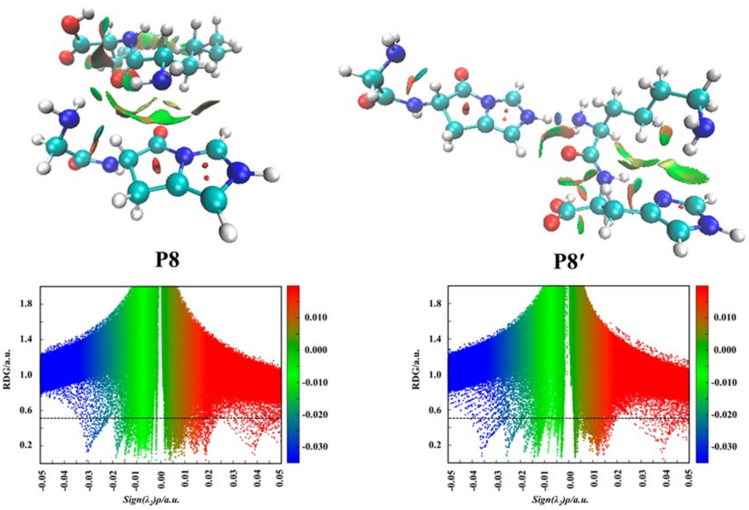

Figure S32. Graphic noncovalent interaction for products (**P8** and **P8′**) obtained from the lactam pathway of protonated GHKH. The upper corresponds to RDG isosurfaces (RDG = 0.5 a.u.); the lower corresponds to scatter maps of RDG vs sign(λ_2)ρ value, where the RDG isosurface of the horizontal line is 0.5 a.u. The color scale bar ranges from -0.035 to 0.02.

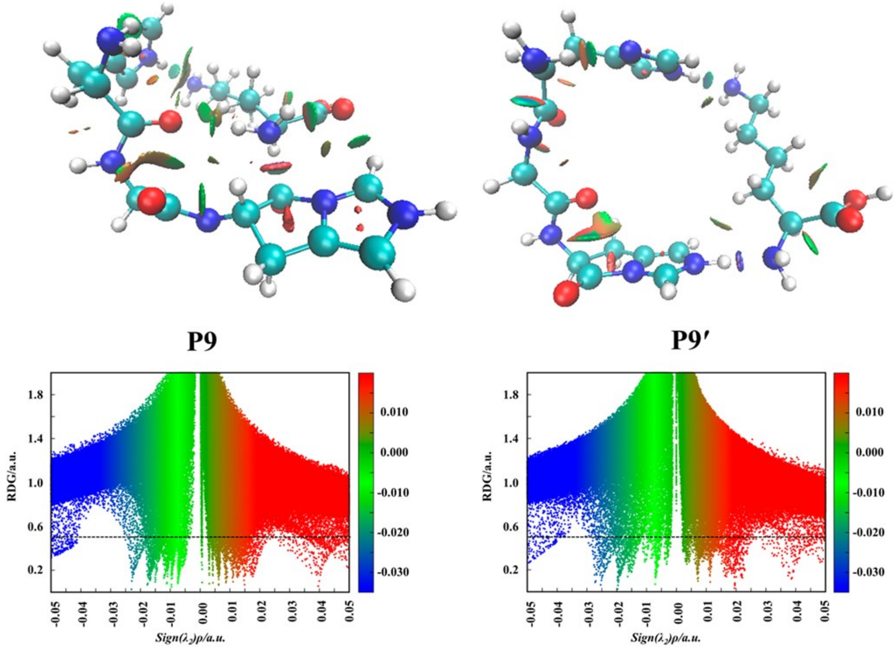

Figure S33. Graphic noncovalent interaction for products (**P9** and **P9′**) obtained from the lactam pathway of protonated HGHK. The upper corresponds to RDG isosurfaces (RDG = 0.5 a.u.); the lower corresponds to scatter maps of RDG vs sign(λ_2)ρ value, where the RDG isosurface of the horizontal line is 0.5 a.u. The color scale bar ranges from -0.035 to 0.02.

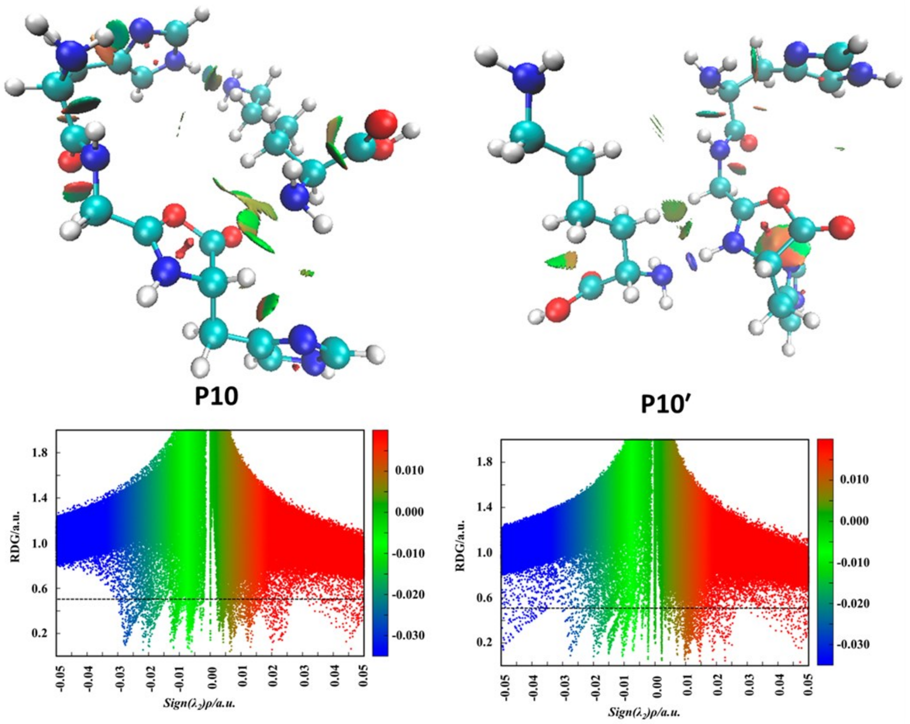

Figure S34. Graphic noncovalent interaction for products (**P10** and **P10′**) obtained from the lactam pathway of protonated HGHK. The upper corresponds to RDG isosurfaces (RDG = 0.5 a.u.); the lower corresponds to scatter maps of RDG vs sign(λ_2)ρ value, where the RDG isosurface of the horizontal line is 0.5 a.u. The color scale bar ranges from -0.035 to 0.02.

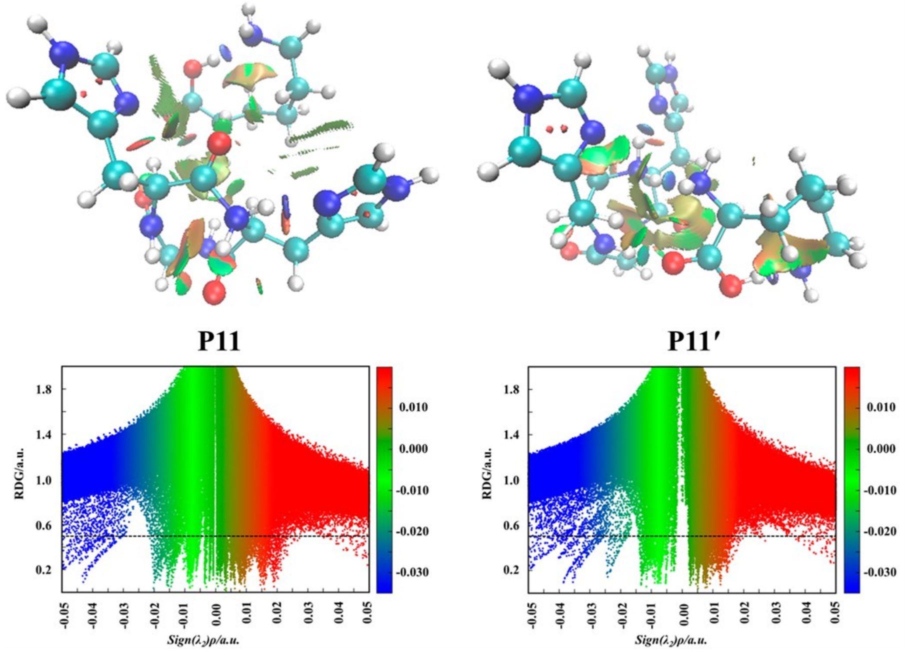

Figure S35. Graphic noncovalent interaction for products (**P11** and **P11′**) obtained from the lactam pathway of protonated HGHK. The upper corresponds to RDG isosurfaces (RDG = 0.5 a.u.); the lower corresponds to scatter maps of RDG vs sign(λ_2)ρ value, where the RDG isosurface of the horizontal line is 0.5 a.u. The color scale bar ranges from -0.035 to 0.02.

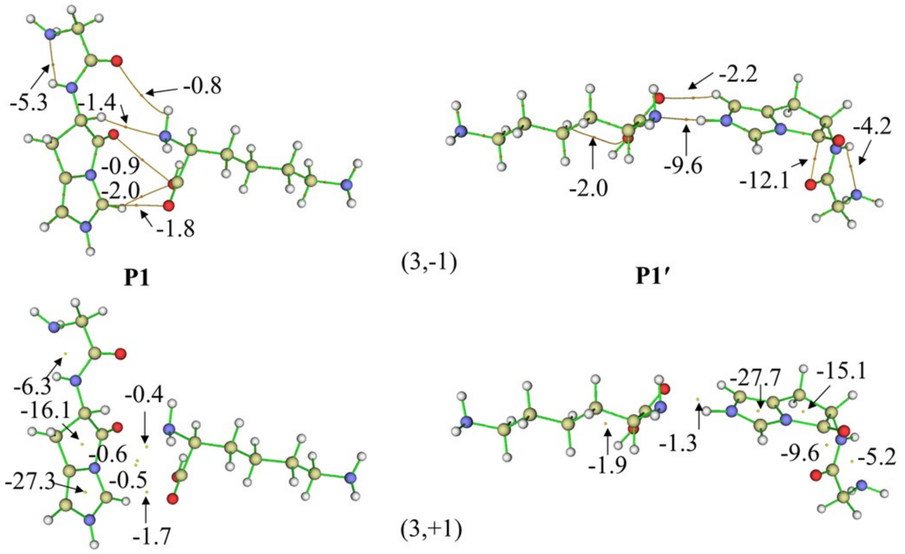

Figure S36. AIM plots of interaction energies based on critical points (CPs) for products **P1** and **P1′**. CPs (3,-1) with orange spheres (upper line), CPs (3,+1) with yellow spheres (lower line), and the bond paths connecting (3,-1) with brown lines.

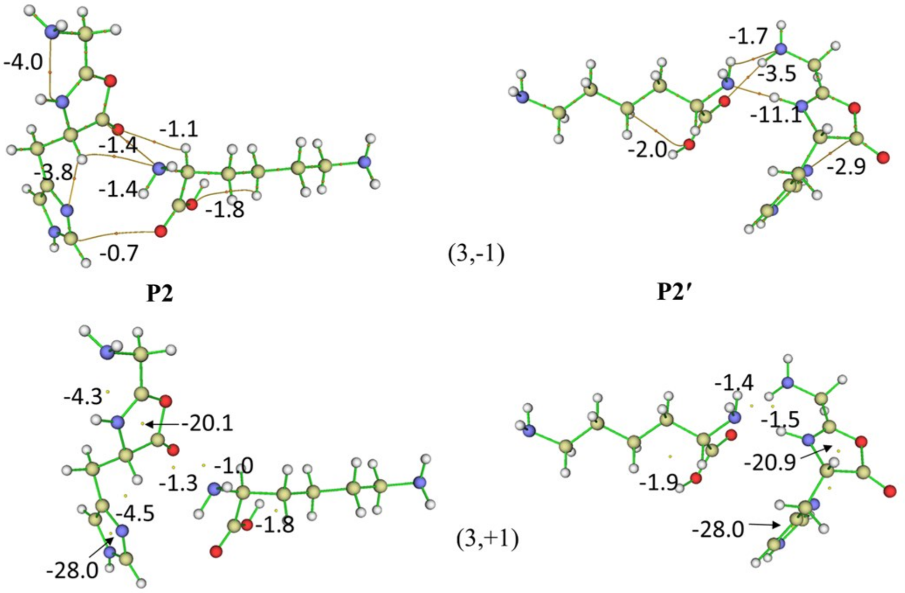

Figure S37. AIM plots of interaction energies based on critical points (CPs) for products **P2** and **P2′**. CPs (3,-1) with orange spheres (upper line), CPs (3,+1) with yellow spheres (lower line), and the bond paths connecting (3,-1) with brown lines.

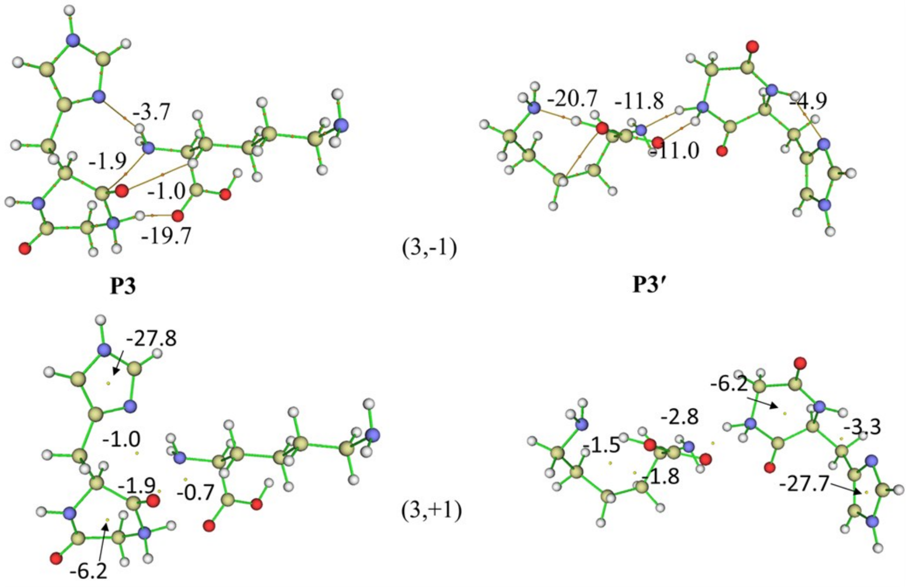

Figure S38. AIM plots of interaction energies based on critical points (CPs) for products **P3** and **P3′**. CPs (3,-1) with orange spheres (upper line), CPs (3,+1) with yellow spheres (lower line), and the bond paths connecting (3,-1) with brown lines.

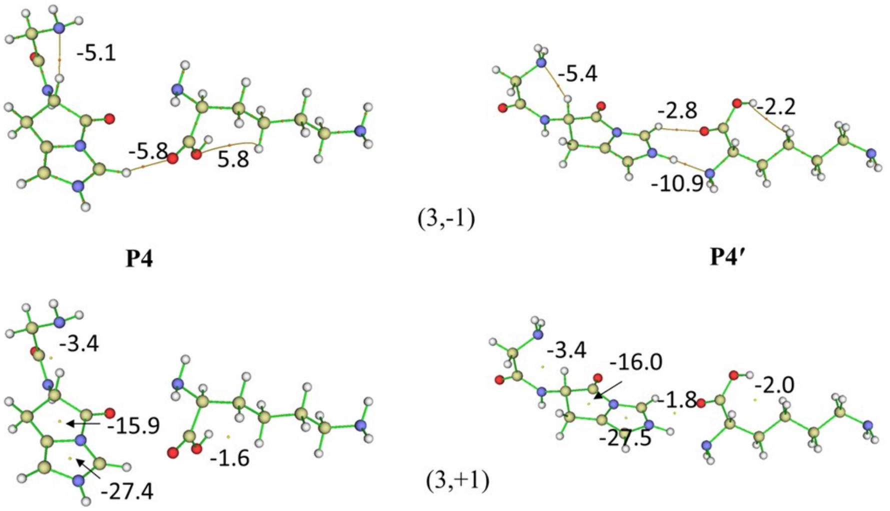

Figure S39. AIM plots of interaction energies based on critical points (CPs) for products **P4** and **P4′**. CPs (3,-1) with orange spheres (upper line), CPs (3,+1) with yellow spheres (lower line), and the bond paths connecting (3,-1) with brown lines.

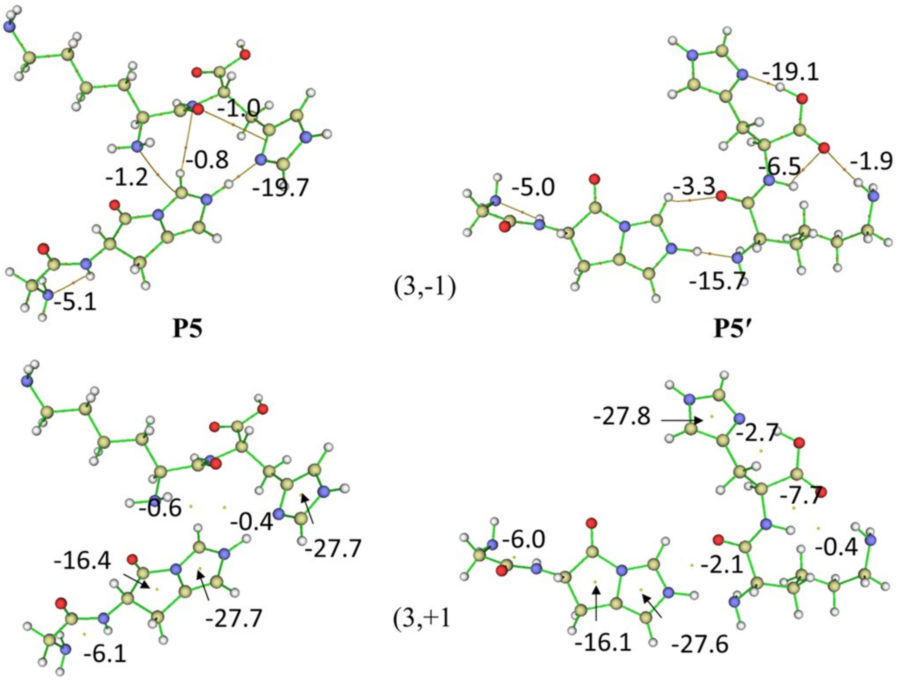

Figure S40. AIM plots of interaction energies based on critical points (CPs) for products **P5** and **P5′**. CPs (3,-1) with orange spheres (upper line), CPs (3,+1) with yellow spheres (lower line), and the bond paths connecting (3,-1) with brown lines.

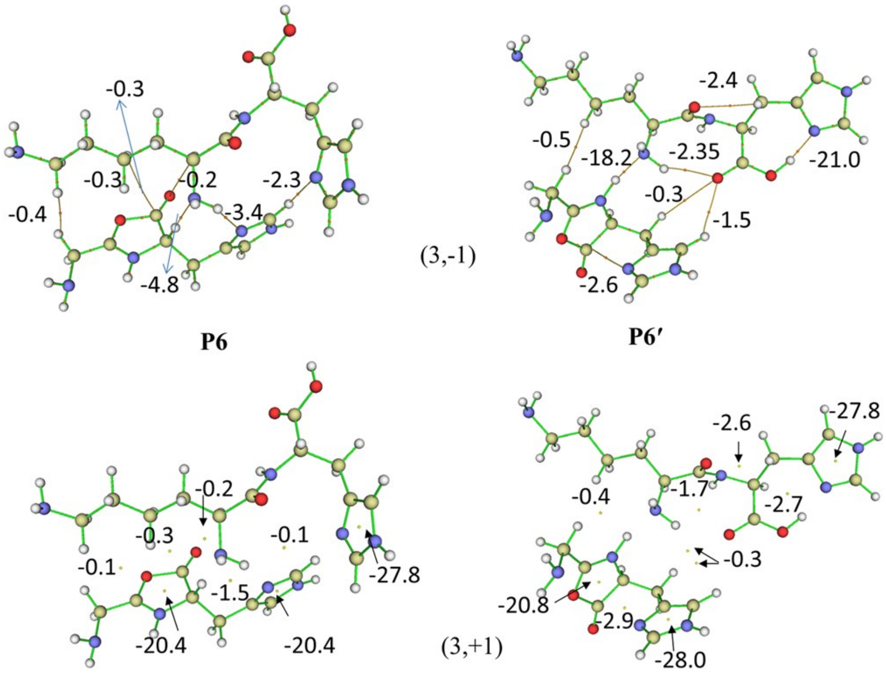

Figure S41. AIM plots of interaction energies based on critical points (CPs) for products **P6** and **P6′**. CPs (3,-1) with orange spheres (upper line), CPs (3,+1) with yellow spheres (lower line), and the bond paths connecting (3,-1) with brown lines.

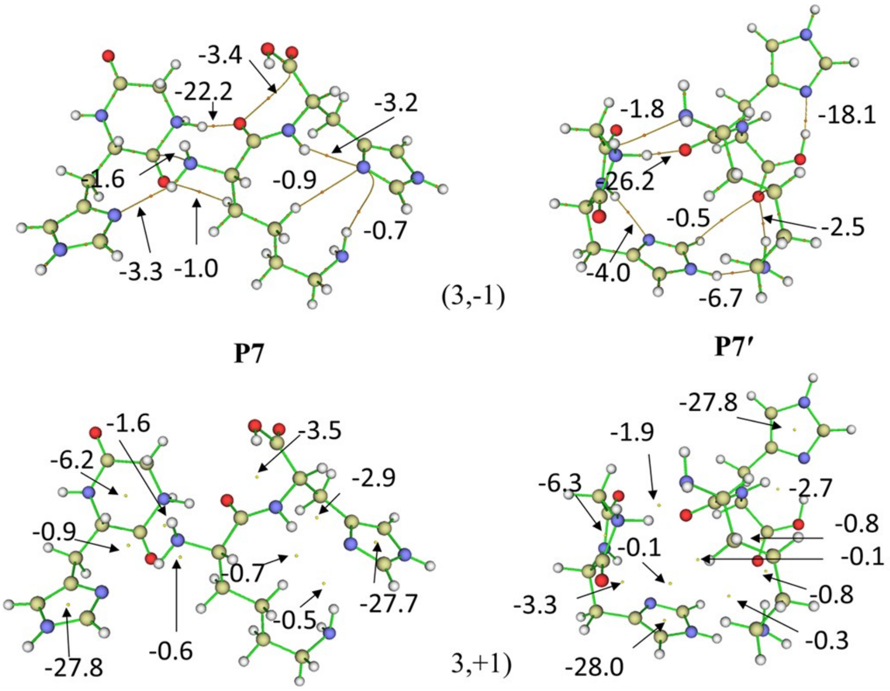

Figure S42. AIM plots of interaction energies based on critical points (CPs) for products **P7** and **P7′**. CPs (3,-1) with orange spheres (upper line), CPs (3,+1) with yellow spheres (lower line), and the bond paths connecting (3,-1) with brown lines.

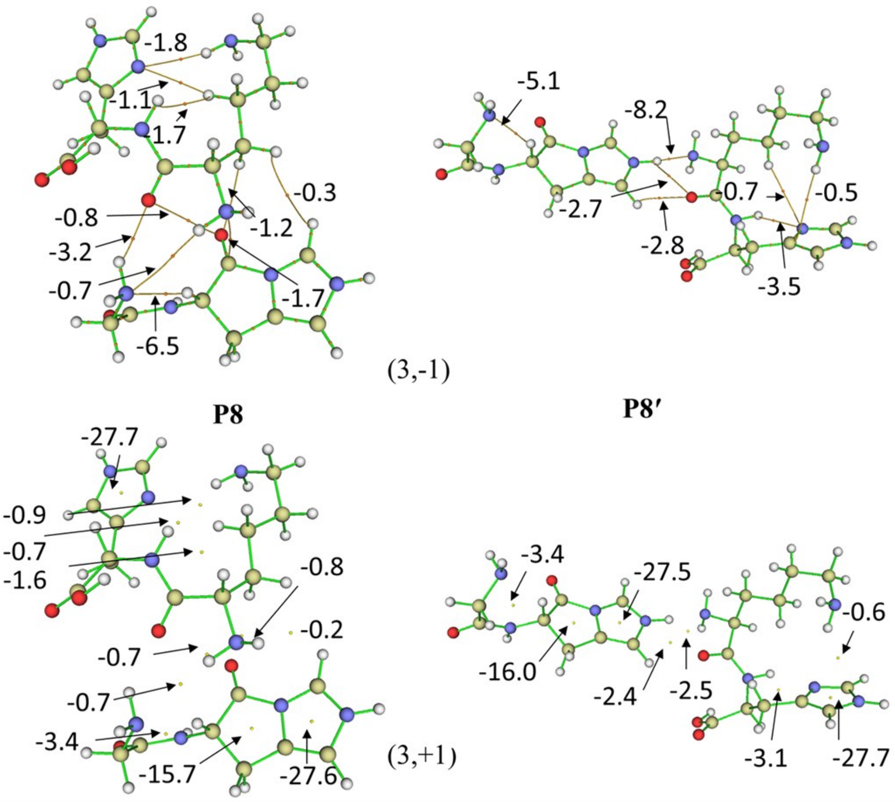

Figure S43. AIM plots of interaction energies based on critical points (CPs) for products **P8** and **P8′**. CPs (3,-1) with orange spheres (upper line), CPs (3,+1) with yellow spheres (lower line), and the bond paths connecting (3,-1) with brown lines.

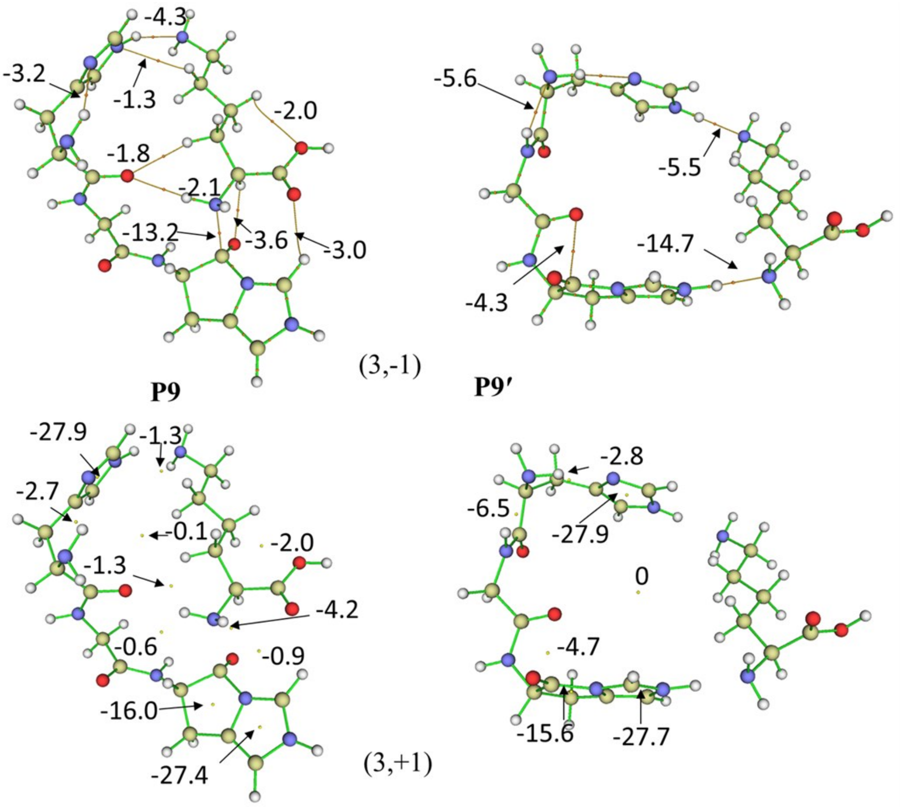

Figure S44. AIM plots of interaction energies based on critical points (CPs) for products **P9** and **P9′**. CPs (3,-1) with orange spheres (upper line), CPs (3,+1) with yellow spheres (lower line), and the bond paths connecting (3,-1) with brown lines.

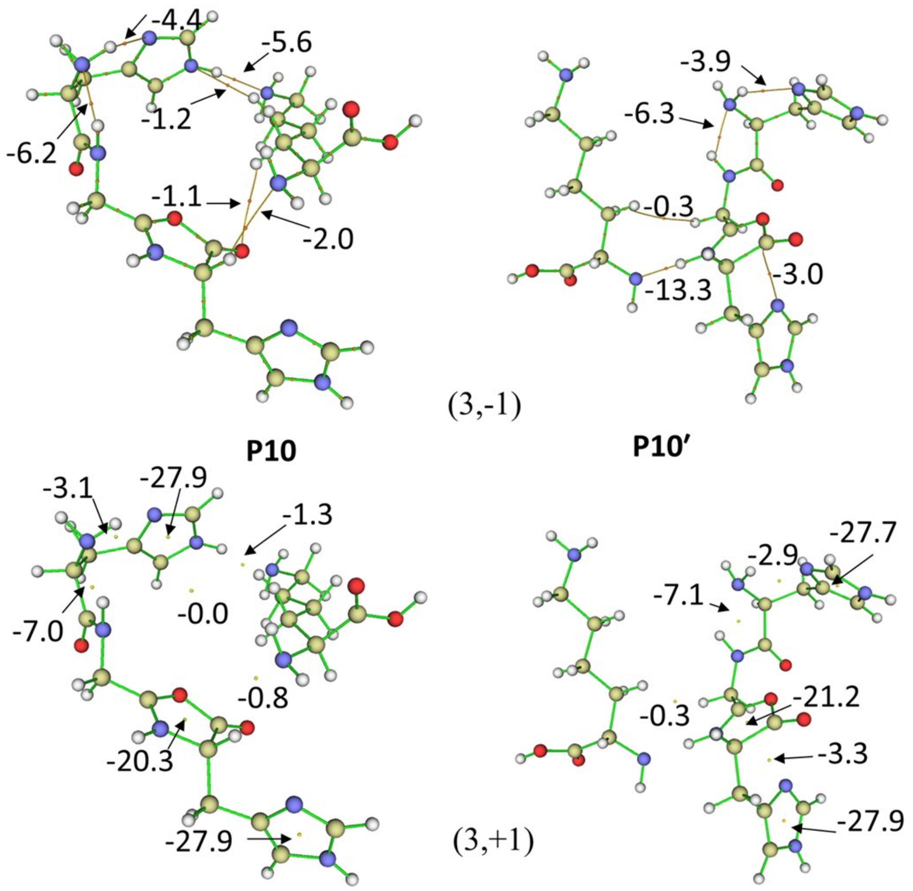

Figure S45. AIM plots of interaction energies based on critical points (CPs) for products **P10** and **P10′**. CPs (3,-1) with orange spheres (upper line), CPs (3,+1) with yellow spheres (lower line), and the bond paths connecting (3,-1) with brown lines.

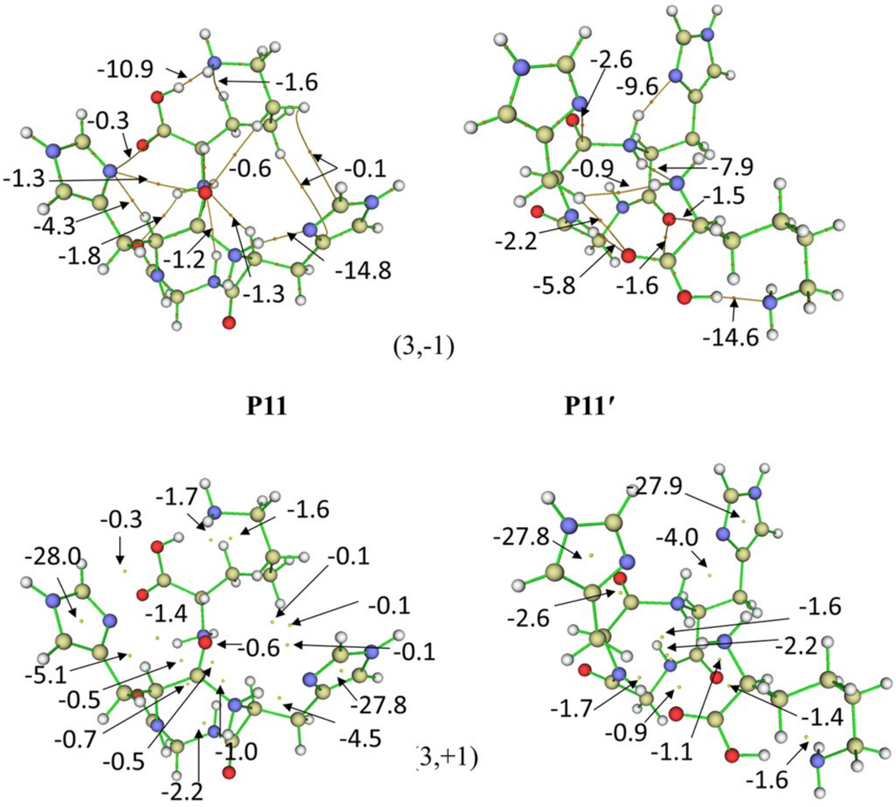

Figure S46. AIM plots of interaction energies based on critical points (CPs) for products **P11** and **P11′**. CPs (3,-1) with orange spheres (upper line), CPs (3,+1) with yellow spheres (lower line), and the bond paths connecting (3,-1) with brown lines.
